# Delayed immune-related adverse events profile associated with immune checkpoint inhibitors: a real-world analysis

**DOI:** 10.3389/fphar.2024.1453429

**Published:** 2024-11-11

**Authors:** Yana Yang, Linman Li, Jing Tian, Linwen Ma, Yaoxin Wu, Qian Luo, Yan Luo

**Affiliations:** ^1^ Nursing Department, The First Affiliated Hospital of Chongqing Medical University, Chongqing, China; ^2^ Health Management Center, The First Affiliated Hospital of Chongqing Medical University, Chongqing, China; ^3^ Radiation Oncology Center, Chongqing University Cancer Hospital, Chongqing, China

**Keywords:** immune checkpoint inhibitors, delayed immune-related adverse events, FAERS, disproportionality analysis, outcomes

## Abstract

**Background:**

Immune-related adverse events (irAEs) typically occur within 3 months of initiating immune-checkpoint inhibitors (ICIs), which has been extensively documented. But the clinical profiles of late-onset irAEs remain inadequately characterized. Therefore, this study aims to quantify the correlation between delayed irAEs and ICIs, and to delineate the profiles of delayed toxicities associated with ICIs using data from the Food and Drug Administration Adverse Event Reporting System (FAERS).

**Methods:**

Data from the January 2011 to December 2023 in FAERS database were extracted. Four signal detection indices, reporting odds ratio (ROR), proportional reporting ratio (PRR), Bayesian confidence propagation neural network (BCPNN) and multi-item gamma Poisson shrinker (MGPS), were employed to evaluate the associations between ICIs and delayed irAEs.

**Results:**

A total of 147,854 cases were included in this study, of which 3,738 cases related to delayed irAEs were identified. Generally, 8 signals at System Organ Class (SOC) level were found to be associated with ICIs. Males had a slightly higher reporting frequencies for respiratory disorders (ROR_975_ = 0.95) and blood and lymphatic system disorders (ROR_025_ = 1.22), but lower reporting frequencies for immune system disorders (ROR_025_ = 1.16). Three monotherapy (anti-PD-1, anti-PD-L1 and anti-CTLA-4) were all associated with significant increasing gastrointestinal disorders (ROR_025_ = 1.66, 1.16, 1.99) and metabolism disorders (ROR_025_ = 2.26, 1.74, 3.13). Anti-PD-1 therapy exhibited higher rates of respiratory toxicities (ROR_025_ = 1.46 versus 0.82) and skin toxicities (ROR_025_ = 1.27 versus 0.94) compared with anti-CTLA-4 therapy. At PT levels, pneumonitis (ROR_025_: from 11.85 to 29.27) and colitis (ROR_025_: from 2.11 to 24.84) were the most notable PT signals associated with all three ICI regimens. For outcomes of delayed irAEs, gastrointestinal disorders showed the highest proportion (51.06%) of death.

**Conclusion:**

Our pharmacovigilance analysis indicates that a small percentage of patients receiving ICIs therapy experience delayed irAEs, which are challenging to manage and may result in severe consequences. Prompt identification and intervention of these delayed irAEs are crucial in clinical practice.

## 1 Introduction

Immune-checkpoint inhibitors (ICIs) have become a key component in the field of cancer therapy, allowing for the potential of long-term survival in patients with challenging malignant tumors, and offering new therapeutic options in (neo)adjuvant and maintenance settings (Johnson et al., 2022). The most widely used targets of ICIs include cytotoxic T lymphocyte-associated antigen-4 (CTLA-4), programmed cell death-1 (PD-1) and anti-programmed cell death ligand-1 (PD-L1) (Martins et al., 2019). Since the approval of the first ICI, ipilimumab, for metastatic melanoma by FDA in 2011, a total of more than 20 different malignancies worldwide have been treated with ICIs (Yin et al., 2023).

One distinguishing feature of ICIs, unlike conventional cancer therapeutic agents, is the potential for sustained responses, even in patients with metastatic solid tumors. However, by inhibiting CTLA4, PD-1 or PD-L1 checkpoints, ICIs can also lead to autoimmune effects known as immune-related adverse events (irAEs) (Singh et al., 2023). While most irAEs occur within the first 3 months of starting immunotherapy, they can also arise at any point during treatment or even months after treatment cessation (Weber et al., 2017). Early irAEs, occurring within 3 months, have been extensively studied (Tao et al., 2023; Zhang et al., 2023). However, the delayed irAEs, defined as those appearing more than 1 year after starting ICIs, have not yet been systematically investigated. Actually, real world data have demonstrated that delayed irAEs may be more frequent in long-term responsers to ICIs and can differ in severity and spectrum from early irAEs. Research by Owen et al. (2021) revealed that 118 melanoma patients treated with ICIs for over 12 months experienced a total of 140 delayed irAEs, with an estimated incidence of 5.3%. The most frequent delayed irAEs included colitis (22%), rash (18%) and pneumonitis (13%). These delayed irAEs are often more severe, distinct from early-onset irAEs, challenging to manage and can be fatal. However, the frequency of delayed irAEs after discontinuing ICIs treatment in a larger patient population, as well as the duration of increased risk for irAEs following immunotherapy cessation, remains unknown.

As the indications of ICIs expands in clinical practice, more patients will be exposed to immunotherapy, potentially leading to life-threatening delayed irAEs. Therefore, it is critical to gather accurate and comprehensive data on the incidence, clinical manifestations, and prognosis of the delayed irAEs from a large patient population. The Food and Drug Administration Adverse Event Reporting System (FAERS) is one of the largest pharmacovigilance databases providing valuable source of real-world data on adverse event, including reports from healthcare professionals, individual patients and drug manufacturers (Zhou et al., 2023). In this study, we aim to analyze the frequency, spectrum and outcomes of the delayed irAEs using FAERS data to enhance our understanding of the safety profiles of ICIs.

## 2 Materials and methods

### 2.1 Study design and data sources

We conducted a pharmacovigilance study on delayed irAEs based on data from the FAERS database spanning from the first quarter of 2011 to the fourth quarter of 2023. The FAERS database includes the following eight types of files: demographic information (DEMO), drug information (DRUG), indications for use (INDI), start and end dates for reported drugs (THER), adverse events (REAC), patient outcomes (OUTC), report sources (RPSR), and invalid reports (DELETED). Keywords used included immune checkpoint inhibitors such as anti-CTLA-4 agents (ipilimumab and tremelimumab), anti-PD-1 agents (nivolumab, pembrolizumab and cemiplimab), and anti-PD-L1 agents (atezolizumab, avelumab and durvalumab). AEs in the FAERS database were coded using preferred terms (PTs) according to the Medical Dictionary for Regulatory Activities (MedDRA) (version 26.1), which is logically structured to contain five levels. PTs are unique descriptors of a single medical concept, such as signs and symptoms and disease diagnosis. A specific PT can be assigned to several high-level terms (HLTs), high-level group terms (HLGTs), and system organ classes (SOCs), which are grouped by aetiology, site of presentation, or purpose. In this study, irAEs were identified using pre-specified list of PTs based on the Society for Immunotherapy of Cancer (SITC), (American Society of Clinical Oncology) ASCO, (European Society for Medical Oncology) ESMO and (National Comprehensive Cancer Network) NCCN guideline/consensus. The PTs of irAEs included in this study are provided in Supplementary Table S1. Cases were defined as a serious medical event if one or more of the following outcomes were reported: death, life-threatening event, hospitalization, disability, congenital anomaly, or other serious medical events.

### 2.2 Data processing procedure

Variables such as Case Identification (CASEID), age, sex, event date, drug names, and outcomes were extracted in each report. Data cleaning was performed prior to analysis with duplicate records removed based on FDA’s recommended method selecting the latest FDA_DT if the CASEID was the same, and choosing the higher PRIMARYID if the CASEID and FDA_DT were the same. In cases where a single patient had multiple reports, the most recent case was retained on the “latest FDA data received to date”. Additionally, the time to onset of irAEs associated with ICIs was calculated as the interval between therapy start date (START_DT) and event onset date (EVENT_DT). Delayed irAEs in this study defined as those with onset >1 year after the initiation of ICIs. Reports were excluded when the START_DT was later than the EVENT_DT or when the report lacked a START_DT or EVENT_DT.

### 2.3 Statistical analysis

We conducted a comprehensive descriptive analysis of the clinical attributes of reports detailing delayed irAEs post-screening, encompassing variables such as gender, age, reporting year, reporting country, clinical outcomes, indication, treatment strategy, and additional clinical characteristics. Adisproportionality analysis was utilized to compare the proportion of specific AEs caused by the target drugs with the proportion of the same AEs in the full database (Zhou et al., 2023). In our study, all drugs in the database were selected as comparisons for the disproportionality approach. Based on the two-by-two contingency table, reporting odds ratio (ROR), proportional reporting ratio (PRR), Bayesian confidence propagation neural network (BCPNN) and multi-item gamma Poisson shrinker (MGPS) were employed to detect an association between various ICI regimens and adverse events in accordance with the disproportionality analysis. The criteria of a significant signal was identified by the 95% confidence interval lower end for ROR (ROR_025_), PRR (PRR_025_), IC (IC_025_) and EBGM (EBGM_05_) (Hauben et al., 2005; Noren et al., 2006; Guo et al., 2022; Jiang et al., 2022). A signal was considered significant if ROR_025_ was greater than 1 with at least 3 cases, PRR value was greater than 2 and Chi-Square was greater than 4, IC_025_ was greater than 0 and EBGM_05_ was greater than 2. Shrinkage transformation was applied to reduce false-negative adverse signals. The equations for the above four algorithms are shown in Supplementary Tables S2, S3. The formula is as follows:

$$\text{ROR}=\frac{\left(a/c\right)}{\left(b/d\right)}=\frac{\text{ad}}{\text{bc}}$$

$$\text{PRR}=\frac{a/\left(a+b\right)}{c/\left(c=d\right)}$$

$$\text{IC}=\log_{2}\frac{a\left(a+b+c+d\right)}{\left(a+b\right)\left(a+c\right)}$$

$$\text{EBGM}=\frac{a\left(a+b+c+d\right)}{\left(a+c\right)\left(a+b\right)}$$

Data were analyzed using SAS version 9.4 (SAS Institute Inc., Cary, NC, United States) and Microsoft Office Excel version 2023 (Microsoft Corp., Redmond, WA, United States).

## 3 Results

### 3.1 Descriptive analysis

**FIGURE 1:**
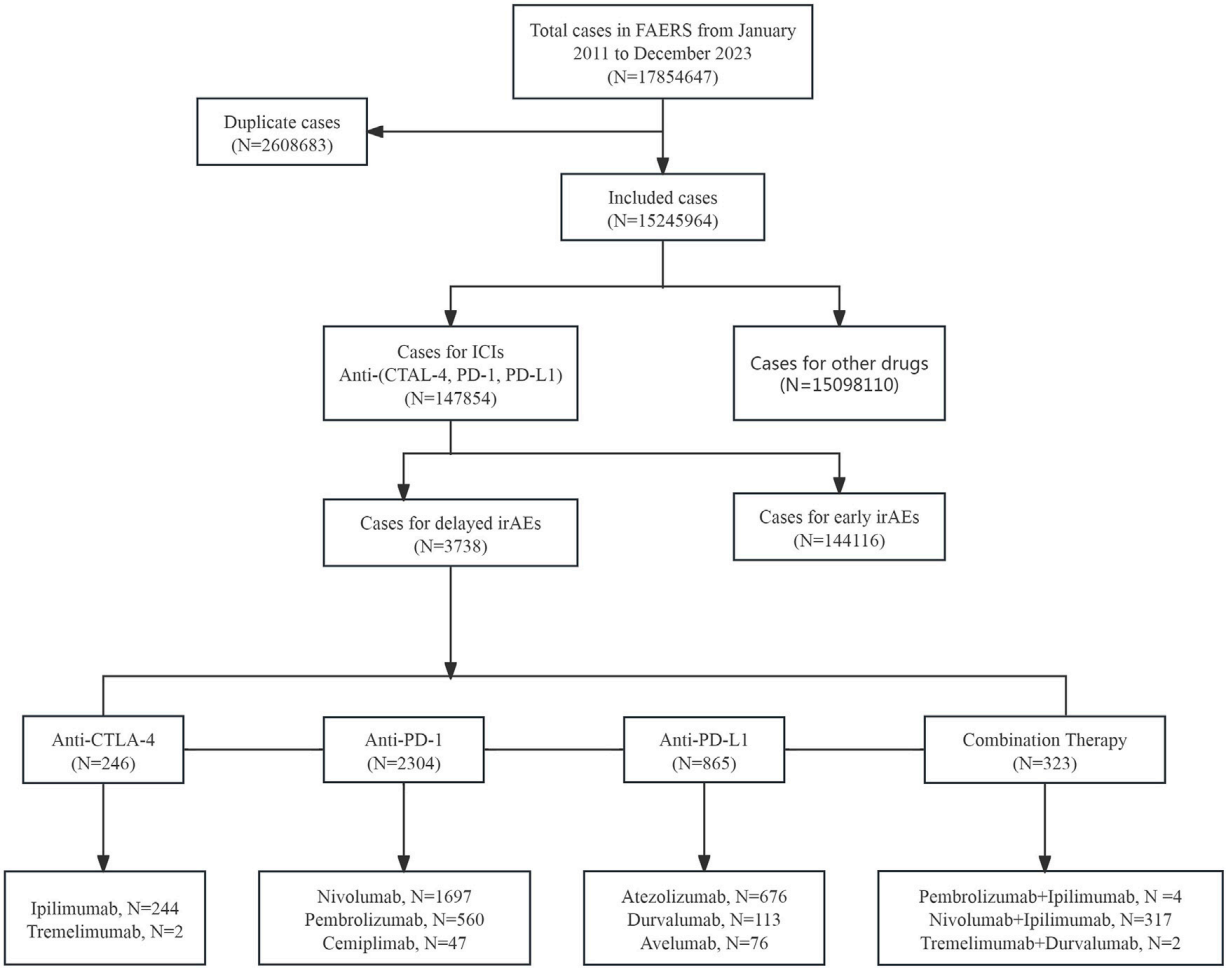
The process of data acquisition and data cleaning from FDA adverse event reporting database (FAERS).

In this study, a total of 17,854,647 cases were extracted from the FAERS database from 2011 to 2023 (Figure 1). After excluding duplicates, the number of cases was 15,245,964, among which 147,854 cases were associated with ICI-related AEs. Additionally, 3,738 cases were found to be associated with delayed irAEs, while 144,116 cases were related to early irAEs following exposure to ICIs.

The clinical characteristics of patients with irAEs were presented in Table 1, including gender, age, reporting year, indications, the distribution of various cancer types and combination therapy strategies. The data indicated that the majority of cases were reported after 2018, reflecting the increased use of ICIs in recent 5 years. Among all irAEs, males accounted for a larger proportion (N = 80,348, 54.34%) than females (N = 50,135, 33.91%). However, delayed irAEs only occurred in 2.31% (3,415/147,854) of all irAEs in the FAERS database. A total of 2,171 cases (63.57%) were reported in male patients, 1,164 cases (34.08%) in female patients, and gender information was not specified for 80 patients. Patients aged 65 years and older represented a larger proportion of delayed irAEs (N = 1947, 57.01%). Physician reported the most cases (N = 1,599, 46.82%), followed by pharmacist (N = 901, 26.38%). The United States reported the highest number of delayed irAEs (N = 776, 22.72%), followed by France (N = 491, 14.38%), Japan (N = 476, 13.94%), and Germany (N = 248, 7.26%). The most commonly reported indication was lung cancer (N = 1,014, 29.69%), followed by malignant melanoma (N = 720, 21.08%) and renal and ureteral cancer (N = 429, 12.56%). Hospitalization was the most frequently reported serious outcome (N = 2,159, 63.22%). Death or life-threatening events occurred in 921 cases (26.977%) of delayed irAEs, indicating the potentially life-threatening nature of delayed irAEs. Among the three categories of ICIs, anti-PD-1 agents were associated with more delayed irAEs (N = 2,304, 67.47%) compared to anti-PD-L1 (N = 865, 25.33%) and anti-CTLA4 (N = 246, 7.20%).

**TABLE 1:** Clinical characteristics of patients with delayed irAEs.

Characteristics	Delayed irAEs (N = 3,415)	All irAEs (N = 147,854)
Gender		
Female	1,164 (34.08%)	50,135 (33.91%)
Male	2,171 (63.57%)	80,348 (54.34%)
Age		
<65	1,338 (42.99%)	44,696 (60.41%%)
≥65	1947 (57.01%)	58,542 (39.59%)
Reporting year		
∼2017	401 (11.74%)	31,268 (21.15%)
2018	295 (8.64%)	15,709 (10.62%)
2019	436 (12.77%)	18,248 (12.34%)
2020	512 (14.99%)	17,787 (12.03%)
2021	563 (16.49%)	18,992 (12.85%)
2022	694 (20.32%)	21,689 (14.67%)
2023	514 (15.05%)	24,161 (16.34%)
Reporter Type		
Consumer	404 (11.83%)	38,728 (26.19%)
Health-professional	495 (14.49%)	18,886 (12.77%)
Pharmacist	901 (26.38%)	27,244 (18.43%)
Physician	1,599 (46.82%)	61,541 (41.62%)
Reporting Countries (top 5)		
USA	776 (22.72%)	57,792 (39.09%)
France	491 (14.38%)	10,736 (7.26%)
Japan	476 (13.94%)	30,954 (20.94%)
Germany	248 (7.26%)	5,823 (3.94%)
Italy	161 (4.71%)	3,184 (2.15%)
Indication (top 5)		
Lung Cancer	1,014 (29.69%)	42,543 (28.77%)
Malignant Melanoma	720 (21.08%)	24,071 (16.28%)
Renal and Ureteric Cancer	429 (12.56%)	12,416 (8.40%)
Hepatobiliary Malignancies	145 (4.25%)	6,629 (4.48%)
Head and Neck Carcinoma	108 (3.16%)	3,941 (2.67%)
Outcome		
Life-Threatening	258 (7.55%)	8,930 (6.04%)
Hospitalization	2,159 (63.22%)	59,077 (39.96%)
Death	663 (19.41%)	37,726 (25.52%)
Other Serious	2,109 (61.76%)	97,872 (66.20%)
Treatment strategy		
Anti-PD-1	2,304 (67.47%)	102,028 (69.01%)
Nivolumab	1,697 (49.69%)	60,636 (41.01%)
Pembrolizumab	560 (16.40%)	39,847 (26.95%)
Cemiplimab	47 (1.38%)	1,545 (1.04%)
Anti-PD-L1	865 (25.33%)	29,484 (19.94%)
Atezolizumab	676 (19.80%)	18,907 (12.79%)
Avelumab	76 (2.23%)	1853 (1.25%)
Durvalumab	113 (3.31%)	8,742 (5.90%)
Anti-CTLA4	246 (7.20%)	16,343 (11.05%)
Ipilimumab	244 (7.14%)	16,243 (10.99%)
Tremelimumab	2 (0.06%)	99 (0.07%)
Combination therapy	323 (9.46%)	14,838 (10.04%)
Pembrolizumab + Ipilimumab	4 (1.24%)	322 (2.17%)
Nivolumab + Ipilimumab	317 (98.14%)	14,449 (97.38)
Tremelimumab + Durvalumab	2 (0.62%)	67 (0.45%)

Among ICIs analyzed in this study, nivolumab had the highest number of cases (N = 1,697, 49.69%) of delayed irAEs, followed by atezolizumab (N = 676, 19.80%) and pembrolizumab (N = 560, 16.40%). In terms of combination therapy, the combination of nivolumab and ipilimumab was associated with the most frequently reported delayed irAEs (N = 317, 98.14%).

### 3.2 Signal of system organ class

**FIGURE 2:**
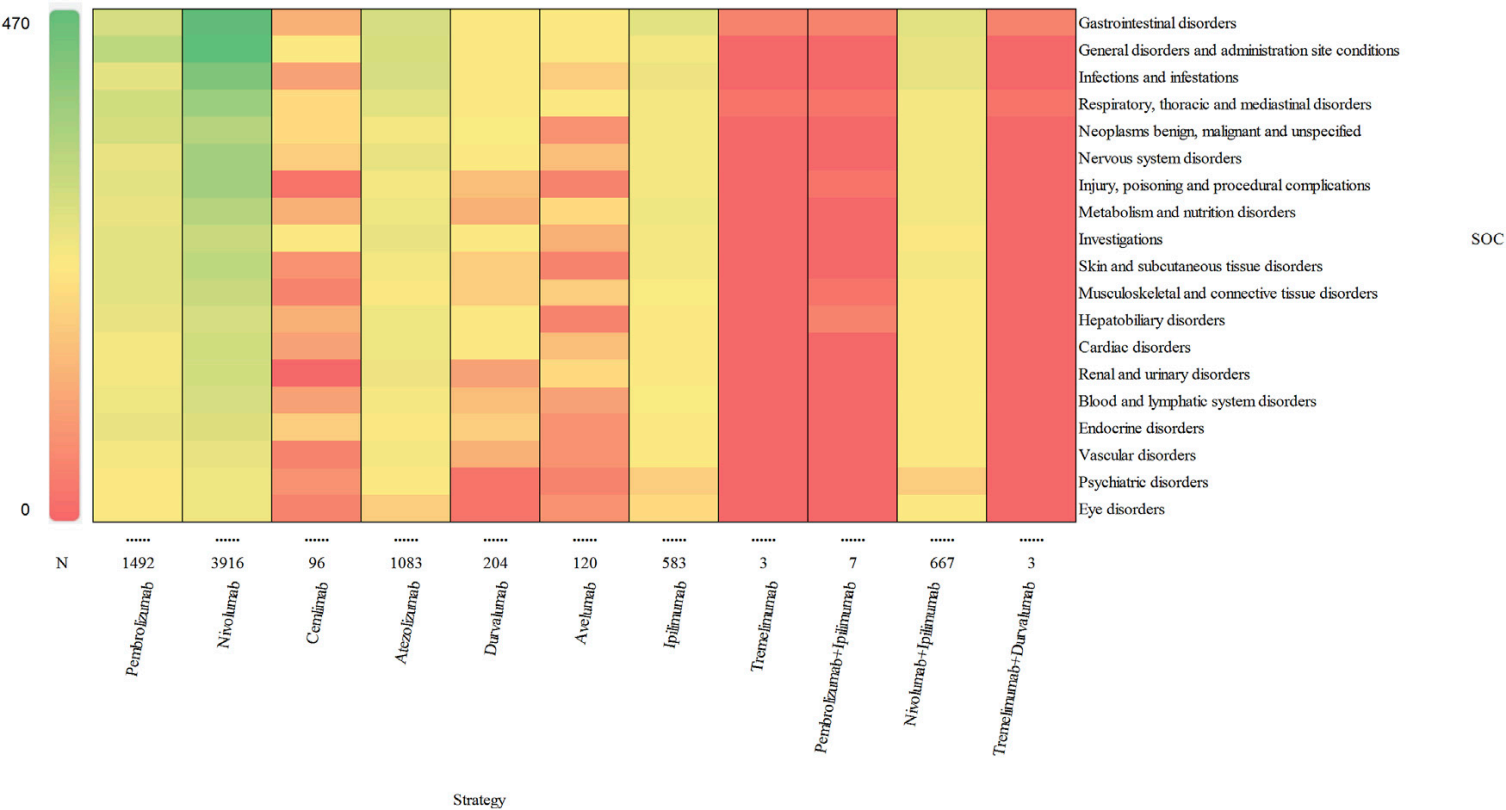
Visualization of reporting cases of delayed irAEs for different treatment strategies at SOC level.

Based on the original data, the signal strength of delayed irAEs at the System Organ Class (SOC) level was described in Supplementary Table S4. We identified delayed irAEs occurring in 27 different SOCs. The reporting cases and types of delayed irAEs at SOC level for various treatment strategies were visualized in Figure 2. Regarding different class-specific ICI regimens, anti-PD-1 drugs (nivolumab, pembrolizumab, and cemiplimab) accounted for the majority of reported delayed irAEs (N = 5,504, 73.42%). Cemiplimab and tremelimumab were approved by the FDA in September 2018 and October 2022, respectively, but were rarely used, leading to limited reporting of delayed irAEs. Among combination therapy, nivolumab + ipilimumab had the highest number of reported delayed irAEs at the SOC level (N = 667, 98.52%), as it was the most commonly used combination regimen in real-world settings.

We identified suspicious signals of ICIs using four pharmacovigilance algorithms (ROR, PRR, BCPNN, and MGPS) and presented the results in Table 2. The significant SOCs associated with ICIs included gastrointestinal disorders (ROR_025_ = 1.30, SOC 10017947), respiratory, thoracic and mediastinal disorders (ROR_025_ = 1.41, SOC 10038738), metabolism and nutrition disorders (ROR_025_ = 2.27, SOC 10027433), skin and subcutaneous tissue disorders (ROR_025_ = 1.17, SOC 10040785), hepatobiliary disorders (ROR_025_ = 2.86, SOC 10019805), renal and urinary disorders (ROR_025_ = 1.42, SOC 10038359), blood and lymphatic system disorders (ROR_025_ = 1.81, SOC 10005329) and endocrine disorders (ROR_025_ = 10.50, SOC 10014698). Among these delayed irAEs, gastrointestinal disorders (N = 842, 11.23%), general disorders and administration site conditions (N = 838, 11.18%), infections and infestations (N = 657, 8.76%) and respiratory, thoracic and mediastinal disorders (N = 583, 7.78%) accounted for two-fifths of the reported adverse events. Notably, the strongest disproportionality association was for endocrine disorders (ROR_025_ = 10.50, χ^2^ = 2074.69, IC_025_ = 3.23, EBGM_05_ = 9.84). Moreover, sex-specific analyses of delayed irAEs at the SOC level were performed. Significant signals were detected in respiratory, thoracic and mediastinal disorders (ROR_975_ = 0.95), hematologic and lymphatic system disorders (ROR_025_ = 1.22), and immune system disorders (ROR_025_ = 1.16), as detailed in Supplementary Table S6.

**TABLE 2:** Signal strength of delayed irAEs at the SOC level in FAERS database.

System organ class (SOC)	Reporting cases	ROR (ROR_025_-ROR_975_)	PRR (χ2)	IC (IC_025_-IC_975_)	EBGM (EBGM_05_-EBGM_95_)
Gastrointestinal disorders	842	1.39 (1.30–1.50)*	1.35 (82.14)*	0.43 (0.32–0.53)*	1.35 (1.25–1.45)
General disorders and administration site conditions	838	0.70 (0.65–0.75)	0.73 (96.64)	−0.45 (-0.55–0.34)	0.73 (0.68–0.79)
Infections and infestations	657	1.06 (0.98–1.15)	1.05 (2.04)	0.08 (-0.04–0.19)	1.05 (0.97–1.14)
Respiratory, thoracic and mediastinal disorders	583	1.53 (1.41–1.67)*	1.49 (99.08)*	0.57 (0.45–0.70)*	1.49 (1.37–1.62)
Neoplasms benign, malignant and unspecified	462	1.99 (1.81–2.19)*	1.93 (211.55)*	0.94 (0.80–1.08)*	1.92 (1.75–2.11)
Nervous system disorders	461	0.70 (0.64–0.77)	0.72 (53.57)	−0.47 (-0.61–0.33)	0.72 (0.66–0.79)
Injury, poisoning and procedural complications	419	0.63 (0.57–0.70)	0.65 (83.61)	−0.61 (-0.75–0.46)	0.65 (0.59–0.72)
Metabolism and nutrition disorders	407	2.51 (2.27–2.77)*	2.43 (346.30)*	1.27 (1.12–1.41)*	2.41 (2.18–2.67)*
Investigations	382	0.79 (0.72–0.88)	0.81 (19.13)	−0.31 (-0.46–0.16)	0.81 (0.73–0.89)
Skin and subcutaneous tissue disorders	356	1.30 (1.17–1.45)*	1.29 (23.85)*	0.36 (0.21–0.52)*	1.29 (1.16–1.43)
Musculoskeletal and connective tissue disorders	303	0.61 (1.54–1.68)	0.62 (72.75)	−0.68 (-0.84–0.51)	0.63 (0.56–0.70)
Hepatobiliary disorders	274	3.23 (2.86–3.65)*	3.15 (401.93)*	1.64 (1.45–1.81)*	3.12 (2.77–3.53)*
Cardiac disorders	263	1.08 (0.96–1.23)	1.08 (1.63)	0.11 (-0.07–0.29)	1.08 (0.95–1.22)
Renal and urinary disorders	260	1.61 (1.42–1.82)*	1.59 (57.27)*	0.66 (0.48–0.84)*	1.58 (1.40–1.79)
Blood and lymphatic system disorders	246	2.06 (1.81–2.34)*	2.03 (129.02)*	1.01 (0.82–1.19)*	2.02 (1.78–2.29)
Endocrine disorders	220	12.04 (10.50–13.80)*	11.71 (2074.69)*	3.50 (3.23–3.63)*	11.28 (9.84–12.94)*
Vascular disorders	167	0.98 (0.84–1.14)	0.98 (0.08)	−0.03 (-0.26–0.20)	0.98 (0.84–1.14)
Psychiatric disorders	98	0.27 (0.22–0.32)	0.28 (196.29)	−1.86 (-2.14–1.56)	0.28 (0.23–0.34)
Eye disorders	93	0.77 (0.63–0.95)	0.77 (6.17)	−0.37 (-0.66–0.06)	0.78 (0.63–0.95)

ROR, Reporting odds ratio; CI, Confidence interval; ROR_025_, The lower limit of 95% CI of the ROR; ROR_975_, The upper limit of 95% CI of the ROR; PRR, Proportional reporting ratio; χ, Chi-squared; IC, Information component; IC_025_, The lower limit of 95% CI of the IC; IC_975_, The upper limit of 95% CI of the IC; EBGM, Empirical Bayesian geometric mean; EBGM_05_, The lower limit of 95% CI of EBGM; EBGM_95_, The upper limit of 95% CI of EBGM. *Indicates statistically significant signals in algorithm.

**FIGURE 3:**
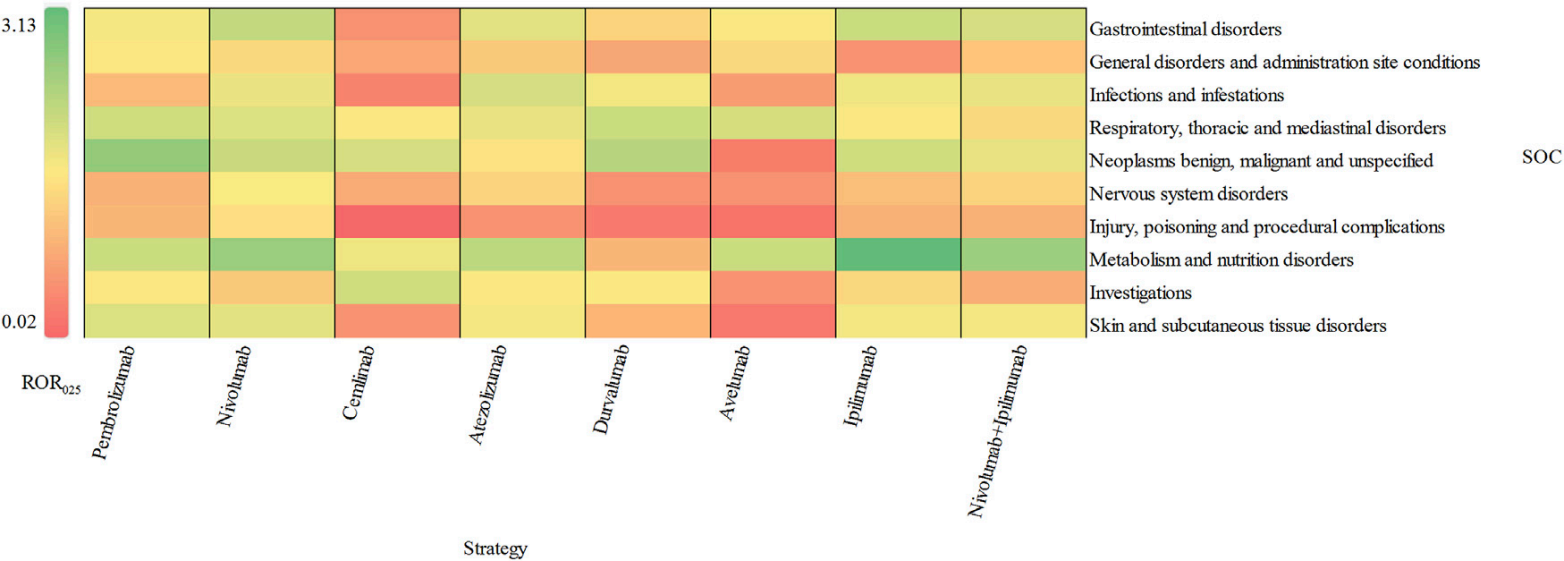
Visualization of delayed irAEs reporting rates for different treatment strategies at SOC level.

The signal values and the association between class-specific ICIs and delayed irAEs are depicted in Figure 3. Among the different class-specific ICI regimens, anti-PD-1 drugs (nivolumab), anti-PD-L1 drugs (atezolizumab) and anti-CTLA-4 drugs (ipilimumab) demonstrated a significant association with gastrointestinal disorders and metabolism disorders. Respiratory system toxicities were significantly associated with anti-PD-1 drugs (pembrolizumab and nivolumab) and anti-PD-L1 drugs (atezolizumab, durvalumab and avelumab) drugs. Only anti-PD-1 drugs (pembrolizumab and nivolumab) exhibited significant signals in skin toxicities, while anti-CTLA-4 drugs did not show a significant association with skin toxicities and respiratory system toxicities. The combination regimen of anti-PD-1 drugs (nivolumab) and anti-CTLA-4 drugs (ipilimumab) did not result in any additional significant signals of delayed irAEs.

### 3.3 Signal of preferred terms

**FIGURE 4:**
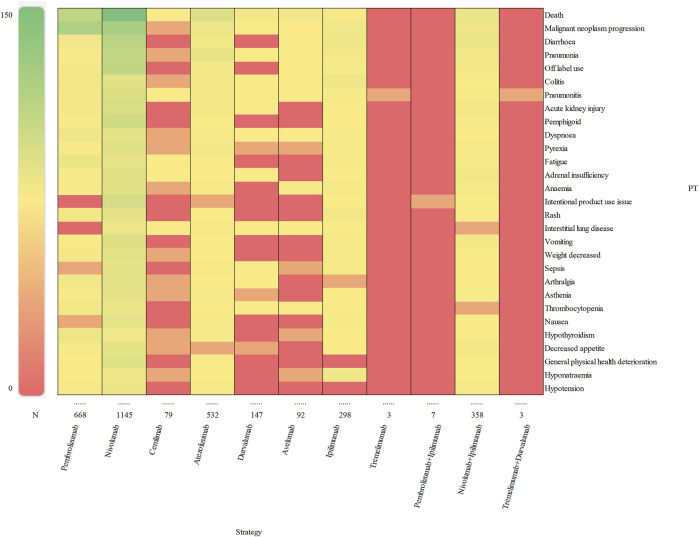
Visualization of reporting cases of delayed irAEs for different treatment strategies at PT level.

We assessed preferred terms (PT) levels in MedDRA for describing the delayed irAEs associated with different ICI regimens. The reported cases and types of delayed irAEs at PT level were visualized in Figure 4. A total of 1648 PT signals were identified from FAERS database, part of which are presented in Supplementary Table S5. Nivolumab exhibited the widest range of PTs among monotherapies, with a total of 1,145 PTs recorded. Analysis of ICIs revealed significant delayed irAEs at the PT level, as shown in Table 3, including but not limited to following PTs: diarrhoea (ROR_025_ = 1.74, PT 10012735), pneumonia (ROR_025_ = 1.29, PT 10035664), colitis (ROR_025_ = 12.81 PT 10009887), pneumonitis (ROR_025_ = 26.45, PT 10035742), acute kidney injury (ROR_025_ = 2.15, PT 10069339), pemphigoid (ROR_025_ = 75.15, PT 10034277), adrenal insufficiency (ROR_025_ = 50.31, PT 10001367), anaemia (ROR_025_ = 1.22, PT 10002034), rash (ROR_025_ = 1.32, PT 10037844) and interstitial lung disease (ROR_025_ = 7.29, PT 10022611). Nivolumab, as one of the most widely used anti-PD-1 drug, exhibited 135 PTs as significant signals that were consistent across four algorithms, ranging from transaminases increased (ROR_025_ = 1.01) to fulminant type 1 diabetes mellitus (ROR_025_ = 320.93). Additionally, 29 PTs were significantly associated with combination treatment regimen of anti-PD-1 drugs (nivolumab) and anti-CTLA-4 drugs (ipilimumab), ranging from general physical health deterioration (ROR_025_ = 1.01) to autoimmune colitis (ROR_025_ = 196.05).

**TABLE 3:** Signal strength of delayed irAEs at PT level.

System organ Class (SOC)	Preferred Terms (PTs)	Reporting Cases	ROR (ROR_025_-ROR_975_)	PRR (χ^2^)	IC (IC_025_-IC_975_)	EBGM (EBGM_05_-EBGM_95_)
Gastrointestinal disorders	Diarrhoea	125	2.08 (1.74–2.48)*	2.06 (68.46)*	1.04 (0.77–1.29)*	2.05 (1.72–2.45)
	Colitis	79	16.09 (12.81–20.21)*	15.93 (1,047.06)*	3.92 (3.35–4.02)*	15.13 (12.05–19)*
	Vomiting	49	1.07 (0.81–1.42)	1.07 (0.25)	0.1 (-0.31–0.51)	1.07 (0.81–1.42)
	Nausea	42	0.70 (0.51–0.94)	0.7 (5.56)	−0.52 (-0.95–0.07)	0.7 (0.52–0.95)
	Abdominal pain	33	0.92 (0.65–1.29)	0.92 (0.25)	−0.12 (-0.62–0.38)	0.92 (0.65–1.29)
Infections and infestations	Pneumonia	116	1.55 (1.29–1.86)*	1.54 (22.16)*	0.62 (0.35–0.88)*	1.54 (1.28–1.85)
	Sepsis	46	2.42 (1.81–3.24)	2.41 (37.84)*	1.26 (0.8–1.64)*	2.4 (1.79–3.21)
	COVID-19	39	0.92 (0.67–1.26)	0.92 (0.26)	−0.12 (-0.57–0.34)	0.92 (0.67–1.26)
	Urinary tract infection	27	0.68 (0.46–0.99)	0.68 (4.12)	−0.56 (-1.09–0.01)	0.68 (0.47–0.99)
	Encephalitis	21	33.9 (21.54–53.33)*	33.81 (596.97)*	4.92 (3.05–4.35)*	30.29 (19.25–47.66)*
Respiratory, thoracic and mediastinal disorders	Pneumonitis	78	33.48 (26.45–42.38)*	33.14 (2,176.32)*	4.9 (4.1–4.79)*	29.76 (23.51–37.67)*
	Dyspnoea	62	0.85 (0.66–1.09)	0.85 (1.61)	−0.23 (-0.59–0.14)	0.85 (0.66–1.09)
	Interstitial lung disease	49	9.70 (7.29–12.90)*	9.64 (367.13)*	3.23 (2.59–3.42)*	9.35 (7.03–12.45)*
	Respiratory failure	29	2.46 (1.71–3.55)*	2.45 (24.83)*	1.29 (0.69–1.75)*	2.44 (1.69–3.52)
	Pulmonary embolism	28	1.38 (0.95–2.00)	1.37 (2.85)	0.46 (-0.1–0.98)	1.37 (0.95–1.99)
Nervous system disorders	Headache	25	0.41 (0.28–0.61)	0.41 (21.25)	−1.28 (-1.81–0.68)	0.41 (0.28–0.61)
	Dizziness	24	0.49 (0.33–0.74)	0.49 (12.51)	−1.02 (-1.56–0.41)	0.49 (0.33–0.74)
	Seizure	22	1.05 (0.69–1.6)	1.05 (0.06)	0.08 (-0.53–0.68)	1.05 (0.69–1.6)
	Cerebral infarction	16	3.39 (2.07–5.55)*	3.38 (26.57)*	1.75 (0.85–2.26)*	3.36 (2.05–5.5)*
	Syncope	15	0.80 (0.48–1.33)	0.8 (0.76)	−0.32 (-1.03–0.42)	0.8 (0.48–1.33)
Metabolism and nutrition disorders	Decreased appetite	41	2.01 (1.47–2.73)*	2 (20.42)*	1 (0.51–1.41)*	1.99 (1.46–2.71)
	Hyponatraemia	39	5.53 (4.03–7.6)*	5.51 (141.24)*	2.44 (1.82–2.75)*	5.42 (3.95–7.45)*
	Diabetic ketoacidosis	37	13.72 (9.86–19.1)*	13.66 (414.25)*	3.71 (2.83–3.79)*	13.08 (9.39–18.2)*
	Hyperglycaemia	32	7.79 (5.48–11.08)*	7.76 (183.64)*	2.92 (2.15–3.17)*	7.58 (5.33–10.78)*
	Dehydration	32	1.75 (1.23–2.47)*	1.74 (10.08)*	0.8 (0.26–1.27)*	1.74 (1.23–2.46)
Skin and subcutaneous tissue disorders	Pemphigoid	68	99.13 (75.15–130.76)*	98.24 (4,853.32)*	6.19 (4.77–5.55)*	73.1 (55.41–96.42)*
	Rash	50	1.75 (1.32–2.31)*	1.74 (15.81)*	0.8 (0.37–1.18)*	1.74 (1.32–2.3)
	Pruritus	37	1.42 (1.02–1.96)*	1.41 (4.47)*	0.5 (0.01–0.95)*	1.41 (1.02–1.95)
	Vitiligo	11	57.47 (30.04–109.95)*	57.39 (506.32)*	5.58 (2.38–4.19)*	47.84 (25.01–91.53)*
	Erythema	9	0.62 (0.32–1.19)	0.62 (2.14)	−0.7 (-1.55–0.27)	0.62 (0.32–1.19)

ROR, Reporting odds ratio; CI, Confidence interval; ROR_025_, The lower limit of 95% CI of the ROR; ROR_975_, The upper limit of 95% CI of the ROR; PRR, Proportional reporting ratio; χ, Chi-squared; IC, Information component; IC_025_, The lower limit of 95% CI of the IC; IC_975_, The upper limit of 95% CI of the IC; EBGM, Empirical Bayesian geometric mean; EBGM_05_, The lower limit of 95% CI of EBGM; EBGM_95_, The upper limit of 95% CI of EBGM. *Indicates statistically significant signals in algorithm.

**FIGURE 5:**
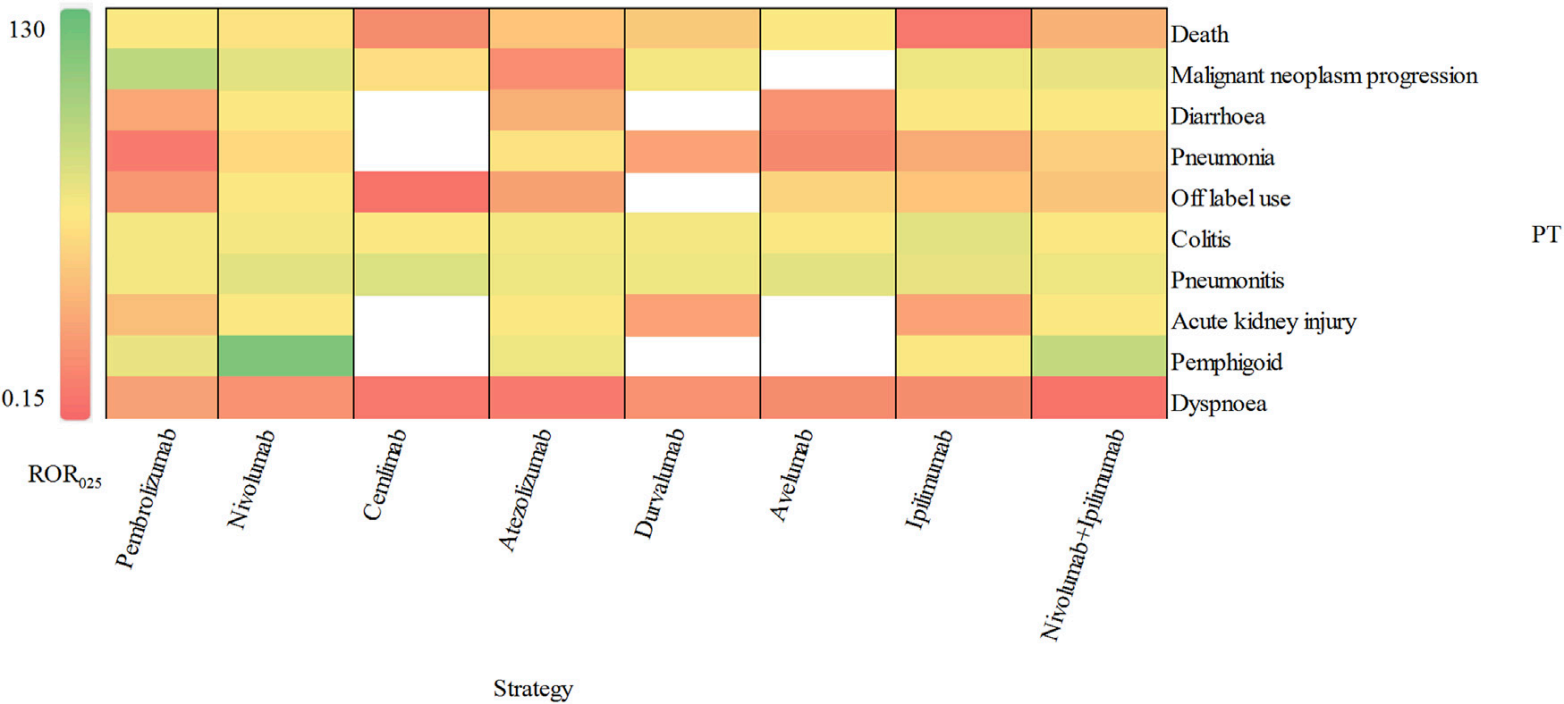
Visualization of delayed irAEs reporting rates for different treatment strategies at PT level.

According to the report, an IC_025_ (the lower limit of 95% CI) value greater than 3.0 indicates a strong signal (Zou et al., 2023). In our study, we identified 10 strong signals at the PT level, including malignant neoplasm progression, colitis, pneumonitis, pemphigoid, adrenal insufficiency, type 1 diabetes mellitus, immune-mediated enterocolitis, hypophysitis, fulminant type 1 diabetes mellitus and encephalitis. Additionally, Figure 5 displays the top 10 most frequently reported PTs of delayed irAEs. Due to the lack of cases or only one case of delayed irAEs recorded for tremelimumab monotherpay, pembrolizumab + ipilimumab and tremelimumab + durvalumab combination therapy, they were excluded from the analysis. It is noteworthy that pneumonitis exhibited the most significant signals across different ICI regimens (ROR_025_: from 11.85 to 29.27), followed by colitis (ROR_025_: from 2.11 to 24.84). Further analysis revealed that nivolumab had most significant signal in pemphigoid (ROR_025_ = 104.75). Interestingly, ipilimumab combined nivolumab showed reduced associations with pneumonitis and colitis.

### 3.4 Outcomes

**FIGURE 6:**
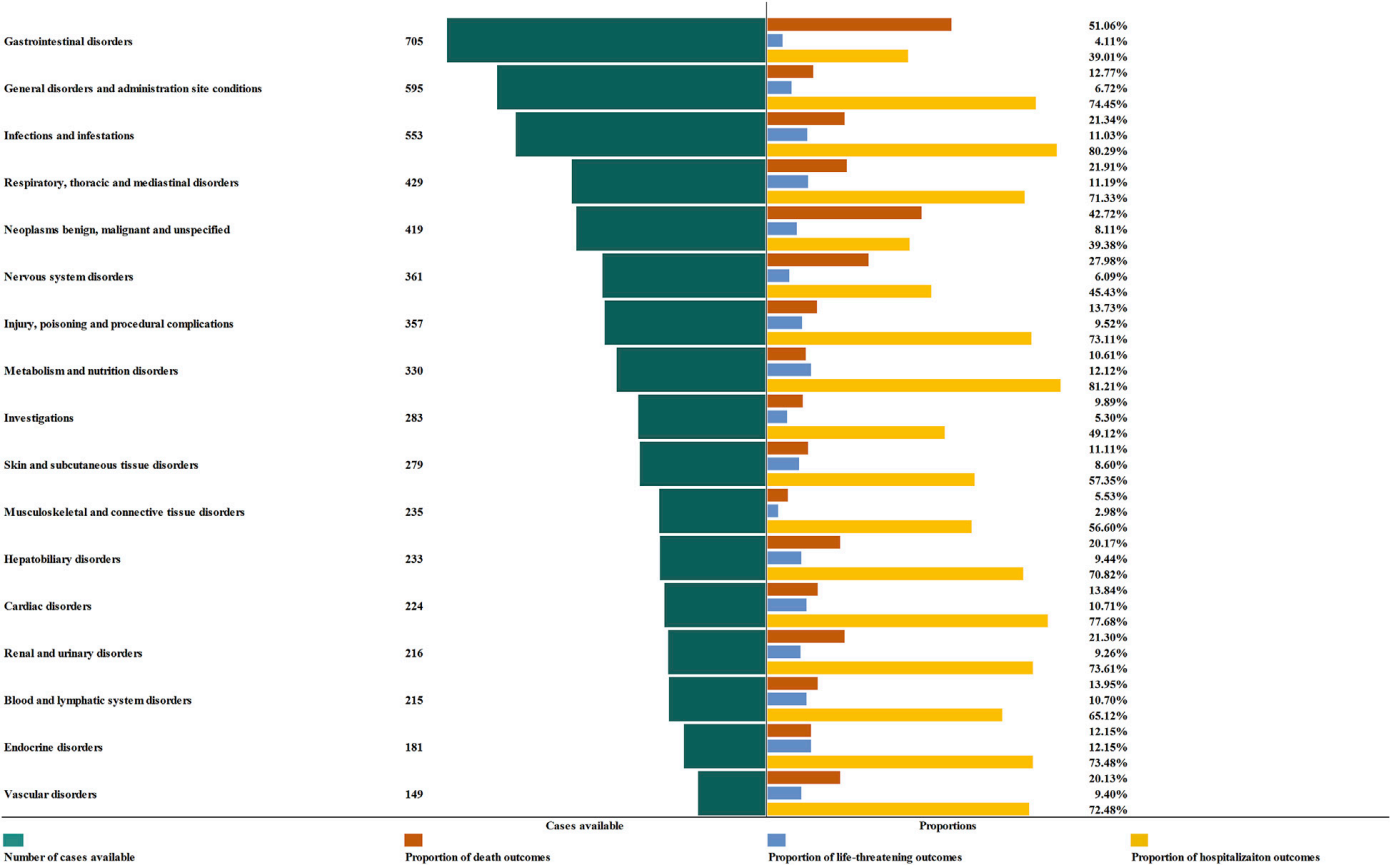
Cases and proportions of different outcomes of irAEs.

In order to improve the prognosis evaluation of delayed irAEs, we examined the proportions of death, life-threatening events, and hospitalization, as shown in Figure 6. Overall, the most severe outcomes of delayed irAEs at the SOC level were reported as death, with the highest proportions in gastrointestinal disorders (51.06%) and endocrine disorders (12.15%) respectively. Additionally, metabolism (12.12%) and respiratory disorders (11.19%) had a higher frequency of life-threatening events compared to other irAEs at SOC level. It is worth noting that the frequencies of hospitalization events were 81.21% for metabolism disorders and 80.29% for infections.

## 4 Discussion

With expanding application in oncology (Livingstone et al., 2022; Lorusso et al., 2024), ICIs have been associated with a higher incidence of irAEs than previously anticipated (Kato et al., 2017; Koyama et al., 2018; Johnson et al., 2022). Unfortunately, delayed irAEs has rarely been documented except for few studies (Owen et al., 2021; Couey et al., 2019). Additionally, details regarding delayed irAEs remain unclear. Therefore, we performed an analysis on delayed ICI-related adverse events using the FAERS database, presenting our findings as follows:

From the first quarter of 2011 to the fourth quarter of 2023, a total of 3,415 cases received ICI monotherapies and 323 cases received combination therapies reported delayed irAEs in our study, which we believe is the largest collection of cases of delayed irAEs to date. The reporting rate for delayed irAEs was approximately 2.31%, which is lower than 5.3% reported by Owen (Owen et al., 2021). This suggests that delayed irAEs are still uncommon. In our descriptive analysis, we observed that males accounted for a higher proportion of delayed irAEs compared to females. This difference may be partly due to the higher incidence of cancer in men, as lung cancer and melanoma were the most commonly reported indications for ICIs. Furthermore, Conforti’s research demonstrated a higher propensity for males to undergo ICI therapy compared to females, attributed to the relatively lower participation rates of women in clinical trials (Conforti et al., 2018). Contrarily, our results contradicted Watson’s analysis (Watson et al., 2019), which employed the World Health Organization (WHO) global database of individual case safety reports, revealing a heightened frequency of adverse drug reactions (ADRs) in females, especially during their reproductive years. Additionally, a separate investigation using the national pharmacovigilance center in the Netherlands found that medications such as thyroid hormones and antidepressants, which had the highest incidence of ADRs, were more frequently reported in women (de Vries et al., 2019).

Actually, to date, few studies have explored sex differences in irAEs, especially delayed irAEs. Extant literature delineates gender-specific disparities in immunological responses to both exogenous and endogenous antigens, highlighting distinct differences in both innate and adaptive immunity between males and females (Klein and Flanagan, 2016). Statistically, females exhibit more robust innate and adaptive immune responses compared to their male counterparts. On the other hand, females constitute approximately 80% of the global patient population suffering from systemic autoimmune diseases (Klein and Flanagan, 2016; Conforti et al., 2018). Consequently, the heightened predisposition of females to autoimmune pathologies could potentially render them more susceptible to irAEs (Menzies et al., 2017). To evaluate the impact of sex on the pharmacovigilance signal for delayed irAEs following ICIs initiation, we conducted further disproportionality analysis. Our results indicated that males had a slightly higher reporting frequencies of delayed irAEs in respiratory disorders (ROR_975_ = 0.95) and blood and lymphatic system disorders (ROR_025_ = 1.22), while females had significant higher reporting frequencies in immune system disorders (ROR_025_ = 1.16). These findings align with studies investigating respiratory toxicity linked to ICIs, which indicated that males exhibited a marginally higher incidence of respiratory system AEs compared to females (ROR = 1.74, 95% CI: 1.70∼1.78) (Cui et al., 2022). This outcome may be partially attributed to greater exposure to cigarette smoke among men (Zhu et al., 2020; Suresh et al., 2018). Regarding hematologic and lymphatic system disorders, our result is different from Li’s study (Li et al., 2023). The specific determinants underlying sex-based disparities are not yet fully elucidated and necessitate additional investigation. No significant signals were detected between male and female patients for other delayed irAEs at the SOC level (Supplementary Table S3). Our findings suggest that sex difference may be an important biological variable for delayed irAEs, although the underlying factors are still unclear and require further investigation in the field of oncology. Furthermore, we observed that patients over 65 years old had a higher reporting frequency of delayed irAEs compared to those under 65 years old. Conversely for all irAEs, patients over 65 years old had much lower reporting rates. The impact of age difference on irAEs, particularly delayed irAEs is not well-established (Baldini et al., 2020; Paderi et al., 2021). and our analysis based on large-scale FAERS data may offer valuable evidence of the associations between age and delayed irAEs. Future studies should pay more attention to age differences in patients with delayed irAEs.

Importantly, we evaluated and compared the incidence of delayed irAEs across various immunotherapy regimens. Overall, there were more reports of delayed irAEs associated with anti-PD-1 inhibitors compared to anti-PD-L1 and anti-CTLA-4 inhibitors. Our analysis revealed that all ICIs demonstrated a higher reporting frequency of metabolism and nutrition disorders as compared to other delayed irAEs at SOC level (ROR_025_: from 0.45 to 3.13), which was consistent with the findings from a previous study (Reese et al., 2020). Moreover, treatment with anti-PD-1 agents exhibited a higher reporting frequency of gastrointestinal disorders in comparison to other ICI regimens (ROR_025_ = 1.66). Conversely, metabolism and nutrition disorders were the most commonly reported delayed irAEs with anti-CTLA-4 medications as opposed to anti-PD-1/anti-PD-L1 medications (ROR_025_ = 3.13). Notably, significant signals for skin and subcutaneous disorders were observed with anti-PD-1 regimens (ROR_025_: from 0.27 to 1.27), suggesting a higher likely-hood of skin toxicities with anti-PD-1 inhibitors. Interestingly, previous study have indicated that combination therapy is associated with increased rates of AEs involving multiple organ systems (Grimaldi et al., 2016). However, our study found that combination of anti-PD-1 and anti-CTLA-4 agents did not appear to further elevate the risk of delayed irAEs, in contrast to what has been reported for early irAEs.

Additionally, our study provides more precise data on the profile of delayed irAEs caused by different ICI regimens at the PT level. A total of 282 significant signals for potential toxicities were identified, including diarrhoea, pneumonia, colitis, pneumonitis, acute kidney injury and pemphigoid. Diarrhoea was more frequently recorded in patients receiving anti-PD-1 and anti-CTLA-4 therapy. Furthermore, we observed that ipilimumab alone or in combination with nivolumab had a higher risk of diarrhoea compared to other ICI regimens. It’s worth noting that we identified two strong signals (IC_025_ > 3.0) for colitis and pneumonitis in all eight monotherapy regimens and one combination regimen. Owen reported that colitis was the most frequent delayed irAEs after adjuvant anti-PD-1 therapy in melanoma patients, which is consistent with our findings (Owen et al., 2021). The prognosis of delayed irAEs was thoroughly examined in our analysis. We observed that death accounted for 51.06% of the gastrointestinal disorders, indicating a significant impact of gastrointestinal complications on patient mortality. The study by Owen (Owen et al., 2021) demonstrated that the delayed gastrointestinal toxicities, such as colitis, increased the mortality rate of melanoma patients. Another severe outcome of delayed irAEs, life-threatening events, represented 12.15% of endocrine disorders. We noted a significant increase in endocrine-related delayed irAEs among female patients with various cancer types. The four of the most frequently recorded delayed irAEs at the PTs level were adrenal insufficiency, hypothyroidism, hypophysitis and hyperthyroidism, which aligns with previous study (Zhai et al., 2019). Given the high incidence of life-threatening events, it is crucial to closely monitor the signs and symptoms of endocrine-related delayed irAEs during ICI therapy.

Notably, delayed irAEs encompass both *de novo* toxicities and recurrences of previous events. Nevertheless, if the interval since the last dose exceeds 1 year, the probability of an alternative etiology increases (Naidoo et al., 2023). Essentially, attributing a re-emergent irAE is relatively straightforward if the patient is still receiving ICI treatment. However, the etiology of autoimmune toxicity emerging months or even years post-discontinuation of ICI treatment remains ambiguous. For example, viral infections may serve as alternative etiologies, potentially causing myocarditis (Rezkalla and Kloner, 2021) and chronic autoimmune conditions such as type 1 diabetes, rheumatoid arthritis and multiple sclerosis (Smatti et al., 2019). To date, clinical data and animal models on delayed or long-term irAEs are insufficient, and it is unclear whether there is a correlation between ICI treatment, intercurrent infections, and the onset of autoimmune disorders. Similar to recurrent irAEs, *de novo* autoimmune conditions should also be considered in differential diagnoses, particularly with the suspicion of an alternative etiology like viral infection. Naidoo et al. (2020) reported that myocarditis or pneumonitis were observed as manifestations that could confound attribution as re-emergent irAEs or *de novo* events arising from infectious etiology. Consequently, the diagnostic certainty of delayed irAEs can be variable, and the potential for misdiagnosis should be acknowledged. Commonly reported confounding factors include the diagnostic misattribution to the sequelae of concomitant chemotherapy, radiotherapy, disease relapse, or septicemia (Couey et al., 2019). Conclusively, comprehensive and detailed data collection from real-world settings to improve the characterization and management of delayed irAEs is necessary.

As a matter of fact, there are several limitations that need to be addressed. Firstly, FAERS is a spontaneous reporting system with multiple sources of data, resulting in inherent constraints such as under reporting, incomplete patient demographic data, nonuniform data format and missing data. Availability of more detailed clinical data could potentially enhance a comprehensive evaluation of the patients’ response rates associated with these irAEs and the durability of their responses.

Secondly, reports in the FAERS database do not require proof of a causal relationship with the drug. The information in the reports only reflects the observations and opinions of the reporters, which makes it impossible to determine whether the reported AEs were indeed caused by the drug. Thirdly, a case report in FAERS could involve several drugs, adverse events, and outcomes, leading to bias in pharmacovigilance analysis. Also, this study did not account for combination chemotherapy, which could have introduced bias into the results. Lastly, the calculation of fatality rates was not feasible due to the lack of comprehensive exposure data, in addition to the fact that mortality may also result from the underlying disease, concomitant irAEs, and other contributory factors. Notwithstanding, our investigation constitutes a comprehensive and meticulous quantification of the potential hazards associated with delayed irAEs in ICIs. These findings may offer critical evidence for subsequent research endeavors and clinical applications.

## 5 Conclusion

Our study systematically and scientifically evaluated the potential hazards using a large datasets from FAERS, outlining a profile of delayed irAEs. These findings provide valuable insights for future investigations and clinical applications in this specific field. In general, delayed irAEs occur in a small subset of cancer patients exposed to ICI regimens, which can be challenging to manage and may result in serious outcomes. Healthcare providers should be aware of the possibility of ICIs causing delayed irAEs, despite their low frequency. It is crucial to educate patients about these potential toxicities before initiating ICI therapy.

## Data Availability

The original contributions presented in the study are included in the article/Supplementary Material, further inquiries can be directed to the corresponding author.
