# Supplementary material for: Delayed immune-related adverse events profile associated with immune checkpoint inhibitors: a real-world analysis
Source: Front Pharmacol. 2024 Nov 11;15:1453429. doi: 10.3389/fphar.2024.1453429 (PMC11586162; doi:10.3389/fphar.2024.1453429)
Supplement: Supplementary file 1 [file DataSheet1.docx]

Supplementary Material

Delayed immune-related adverse event profile associated with immune checkpoint inhibitors: a real-world analysis

Yana Yang^1^, Linman Li^2^, Jing Tian^2^, Linwen Ma^2^, Yaoxin Wu^2^, Qian Luo^3^, Yan Luo^3*^

*** Correspondence:** Yan Luo: yanluo2018@cqu.edu.cn

# Supplementary Tables

| **PTs of irAEs** |
| --- |
| pruritus, pruritus generalised, vitiligo, dermatitis, dermatitis acneiform, rash maculo-papular, rash papular, rash erythematous, rash generalised, rash macular, rash morbilliform, rash pruritic, rashpustular, rash vesicular, erythema multiforme, dermatitis bullous, dermatitis exfoliative, dermatitis exfoliative generalised, dermatitis psoriasiform, autoimmune dermatitis, diarrhoea, colitis, autoimmune colitis, colitis ulcerative, colitis ischaemic, colitis microscopic, enterocolitis, enterocolitis haemorrhagic, primary hypothyroidism, hypothyroidism, autoimmune hypothyroidism, hyperthyroidism, thyroiditis, autoimmune thyroiditis, hypophysitis, lymphocytic hypophysitis, primary adrenal insufficiency, type 1 diabetes mellitus, fulminant type 1 diabetes mellitus, hepatitis, autoimmune hepatitis, hepatitis acute, hepatitis fulminant, pneumonitis, acute interstitial pneumonitis, myositis, polymyositis, polymyalgia rheumatica, nephritis, autoimmune nephritis tubulointerstitial nephritis, myasthenia gravis, guillain-barre syndrome, neuropathy peripheral, autonomic neuropathy, meningitis aseptic, encephalitis, myelitis transverse, autoimmune neuropathy, encephalitis autoimmune, limbic encephalitis, myelitis, encephalomyelitis, immune thrombocytopenic purpura, myocarditis, autoimmune myocarditis, pericarditis, pericarditis malignant, uveitis, autoimmune uveitis, iritis, episcleritis, arthritis, autoimmune arthritis, arthritis reactive, polyarthritis, rheumatoid arthritis, seronegative arthritis, aplastic anaemia, autoimmune haemolytic anaemia, vogt-koyanagi-harada syndrome, pemphigoid, conjunctivitis, leukoderma, psoriasis, pancreatitis, pancreatitis acute, autoimmune pancreatitis, histiocytosis haematophagic, sjogren's syndrome, sialoadenitis, cytokine release syndrome, synovitis, latent autoimmune diabetes in adults, stevens-johnson syndrome, toxic epidermal necrolysis, autoimmune pancytopenia, immune-mediated adverse reaction, iridocyclitis, autoimmune disorder, noninfective encephalitis, haemolytic uraemic syndrome, blepharitis, acquired haemophilia, radiation pneumonitis |

**Supplementary Table 1 Summary of Preferred Terms (PTs) of irAEs**

| **Item** | **Target adverse events reported** | **Other adverse events reported** | **Total** |
| --- | --- | --- | --- |
| **Target drugs** | a | b | a + b |
| **Other drugs** | c | d | c + d |
| **Total** | a + c | b + d | a + b + c + d |

**Supplementary Table 2 Two-by-two contingency table for disproportionality analysis**

| **Method** | **Calculation formula** | **﻿Criteria** |
| --- | --- | --- |
| ROR | $ROR=\frac{a / c}{b / d}$ | a ≥ 3  ROR ≥ 1  95%CI (lower limit) > 1 |
|  | $SE(lnROR)=\sqrt{\frac{1}{a}+\frac{1}{b}+\frac{1}{c}+\frac{1}{d}}$ |  |
|  | $95\%CI= e^{\ln\left( ROR \right)\pm1.96se}$ |  |
| PRR | $PRR=\frac{a / (a+b)}{c / (c+d)}$ | a ≥ 3  PRR ≥ 2  95%CI (lower limit) > 1 |
|  | $SE(lnPRR)=\sqrt{\frac{1}{a}-\frac{1}{a+b}+\frac{1}{c}-\frac{1}{c+d}}$ |  |
|  | $95\%CI= e^{\ln\left( PRR \right)\pm1.96se}$ |  |
|  | $\chi2 =\frac{{(ad-bc)}^{2}(a+b+c+d)}{( a+b)(a+c)(c+d)(b+d)}$ | a ≥ 3  PRR ≥ 2  $\chi2\geq4$ |
| BCPNN | IC=${log}_{2}\frac{p(x,y)}{p(x)p(y)}={log}_{2}\frac{a(a+b+c+d)}{(a+b)(a+c)}$ | IC_025_>0 |
|  | E(IC)=${log}_{2}\frac{(a+\gamma11)(a+b+c+d+\alpha)(a+b+c+d+\beta)}{（a+b+c+d+\gamma）(a+b+\alpha1)(a+c+\beta1)}$ |  |
|  | $V\left( IC \right)=\frac{1}{{(ln2)}^{2}}\{\left[ \frac{\left( a+b+c+d \right)-a+\gamma-\gamma11}{\left( a+\gamma11 \right)\left( 1+a+b+c+d+\gamma\right)} \right]+\left[ \frac{\left( a+b+c+d \right)-\left( a+b \right)+\alpha-\alpha1}{\left( a+b+\alpha1 \right)\left( 1+a+b+c+d+\alpha\right)} \right]+\left[ \frac{\left( a+b+c+d \right)-\left( a+c \right)+\beta-\beta1}{\left( a+c+\beta1 \right)\left( 1+a+b+c+d+\beta\right)} \right]\}$ |  |
|  | $\gamma=\gamma11\frac{(a+b+c+d+\alpha)(a+b+c+d+\beta)}{(a+b+\alpha1)(a+c+\beta1)}$ |  |
|  | *IC-2SD=E(IC)-2*$\sqrt{V(IC)}$  $\alpha1=\beta1=1；\alpha=\beta=2；\gamma11=1$ |  |
| EBGM | $EBGM=\frac{a(a+b+c+d)}{\left( a+c \right)(a+b)}$ | EBGM_05_>2 |
|  | $SE(lnEBGM)=\sqrt{\frac{1}{a}+\frac{1}{b}+\frac{1}{c}+\frac{1}{d}}$ |  |
|  | $95\%CI= e^{\ln\left( EBGM \right)\pm1.96se}$ |  |
| IC | $\begin{aligned} IC=\log_{2}\left( \frac{N_{\mathrm{observed}}+ 0.5}{N_{\mathrm{expected}}+ 0.5} \right) \end{aligned}$ |  |
|  | $\begin{aligned} N_{\mathrm{expected}}=\frac{\left( N_{\mathrm{drug}}*N_{\mathrm{effect}} \right)}{N_{\mathrm{total}}} \end{aligned}$ |  |
|  | $\begin{aligned} \mathrm{IC}_{025}=\log_{2}\left( \frac{N_{\mathrm{observed}}+ 0.5}{N_{\mathrm{expected}}+ 0.5} \right)-3.3*\left( N_{\mathrm{observed}}+0.5 \right)^{-\frac{1}{2}}-2*\left( N_{\mathrm{observed}}+0.5 \right)^{-\frac{3}{2}} \end{aligned}$ |  |
|  | $\begin{aligned} \mathrm{IC}_{975}=\log_{2}\left( \frac{N_{\mathrm{observed}}+ 0.5}{N_{\mathrm{expected}}+ 0.5} \right)+2.4*\left( N_{\mathrm{observed}}+0.5 \right)^{-\frac{1}{2}}-0.5*\left( N_{\mathrm{observed}}+0.5 \right)^{-\frac{3}{2}} \end{aligned}$ |  |
|  | N_expected_: the number of case reports expected for the drug-ADR pairs.  N_observed_: the actual number of case reports for the drug-ADR pairs.  N_effect_: the number of case reports for the ADR, regardless of the drug.  N_total_: the total number of case reports in the database.  N_drug_: the number of case reports for the drug, regardless of the ADR. | |

**Supplementary Table 2 The principles of disproportionate measurement and the criteria for signal detection**

| **System Organ Class (SOC)** | **Reporting  Cases** | **ROR (ROR_025_-ROR_975_)** | **PRR (χ^2^)** | **IC (IC_025_-IC_975_)** | **EBGM (EBGM_05_-EBGM_95_)** |
| --- | --- | --- | --- | --- | --- |
| Gastrointestinal disorders | 842 | 1.39(1.30-1.50)* | 1.35(82.14)* | 0.43(0.32-0.53)* | 1.35(1.25-1.45) |
| General disorders and administration site conditions | 838 | 0.70(0.65-0.75) | 0.73(96.64) | -0.45(-0.55--0.34) | 0.73(0.68-0.79) |
| Infections and infestations | 657 | 1.06(0.98-1.15) | 1.05(2.04) | 0.08(-0.04-0.19) | 1.05(0.97-1.14) |
| Respiratory, thoracic and mediastinal disorders | 583 | 1.53(1.41-1.67)* | 1.49(99.08)* | 0.57(0.45-0.70)* | 1.49(1.37-1.62) |
| Neoplasms benign, malignant and unspecified | 462 | 1.99(1.81-2.19)* | 1.93(211.55)* | 0.94(0.80-1.08)* | 1.92(1.75-2.11) |
| Nervous system disorders | 461 | 0.70(0.64-0.77) | 0.72(53.57) | -0.47(-0.61--0.33) | 0.72(0.66-0.79) |
| Injury, poisoning and procedural complications | 419 | 0.63(0.57-0.70) | 0.65(83.61) | -0.61(-0.75--0.46) | 0.65(0.59-0.72) |
| Metabolism and nutrition disorders | 407 | 2.51(2.27-2.77)* | 2.43(346.30)* | 1.27(1.12-1.41)* | 2.41(2.18-2.67)* |
| Investigations | 382 | 0.79(0.72-0.88) | 0.81(19.13) | -0.31(-0.46--0.16) | 0.81(0.73-0.89) |
| Skin and subcutaneous tissue disorders | 356 | 1.30(1.17-1.45)* | 1.29(23.85)* | 0.36(0.21-0.52)* | 1.29(1.16-1.43) |
| Musculoskeletal and connective tissue disorders | 303 | 0.61(1.54-1.68) | 0.62(72.75) | -0.68(-0.84--0.51) | 0.63(0.56-0.70) |
| Hepatobiliary disorders | 274 | 3.23(2.86-3.65)* | 3.15(401.93)* | 1.64(1.45-1.81)* | 3.12(2.77-3.53)* |
| Cardiac disorders | 263 | 1.08(0.96-1.23) | 1.08(1.63) | 0.11(-0.07-0.29) | 1.08(0.95-1.22) |
| Renal and urinary disorders | 260 | 1.61(1.42-1.82)* | 1.59(57.27)* | 0.66(0.48-0.84)* | 1.58(1.40-1.79) |
| Blood and lymphatic system disorders | 246 | 2.06(1.81-2.34)* | 2.03(129.02)* | 1.01(0.82-1.19)* | 2.02(1.78-2.29) |
| Endocrine disorders | 220 | 12.04(10.50-13.80)* | 11.71(2074.69)* | 3.50(3.23-3.63)* | 11.28(9.84-12.94)* |
| Vascular disorders | 167 | 0.98(0.84-1.14) | 0.98(0.08) | -0.03(-0.26-0.20) | 0.98(0.84-1.14) |
| Psychiatric disorders | 98 | 0.27(0.22-0.32) | 0.28(196.29) | -1.86(-2.14--1.56) | 0.28(0.23-0.34) |
| Eye disorders | 93 | 0.77(0.63-0.95) | 0.77(6.17) | -0.37(-0.66--0.06) | 0.78(0.63-0.95) |
| Surgical and medical procedures | 44 | 0.27(0.2-0.36) | 0.27(87.00) | -1.87(-2.28--1.41) | 0.27(0.20-0.37) |
| Immune system disorders | 39 | 0.9(0.66-1.24) | 0.9(0.41) | -0.15(-0.6-0.31) | 0.9(0.66-1.24) |
| Pregnancy, puerperium and perinatal conditions | 26 | 0.62(0.42-0.91) | 0.62(6.03) | -0.69(-1.22--0.11) | 0.62(0.42-0.91) |
| Ear and labyrinth disorders | 25 | 0.78(0.52-1.15) | 0.78(1.59) | -0.36(-0.92-0.22) | 0.78(0.53-1.15) |
| Reproductive system and breast disorders | 16 | 0.2(0.13-0.33) | 0.21(49.22) | -2.27(-2.90--1.50) | 0.21(0.13-0.34) |
| Product issues | 9 | 0.09(0.05-0.17) | 0.09(83.19) | -3.46(-4.24--2.41) | 0.09(0.05-0.17) |
| Social circumstances | 5 | 0.14(0.06-0.34) | 0.14(26.13) | -2.82(-3.77--1.41) | 0.14(0.06-0.34) |
| Congenital, familial and genetic disorders | 2 | 0.1(0.02-0.38) | 0.1(17.03) | -3.37(-4.52--1.19) | 0.1(0.02-0.39) |

**Supplementary Table 3** Signal strength of delayed irAEs at Organ System Class (SOC). ROR, Reporting odds ratio; CI, Confidence interval; ROR_025_, The lower limit of 95% CI of the ROR; ROR_975_, The upper limit of 95% CI of the ROR; PRR, Proportional reporting ratio; χ, Chi-squared; IC, Information component; IC_025_, The lower limit of 95% CI of the IC; IC_975_, The upper limit of 95% CI of the IC; EBGM, Empirical Bayesian geometric mean; EBGM_05_, The lower limit of 95% CI of EBGM; EBGM_95_, The upper limit of 95% CI of EBGM. *Indicates statistically significant signals in algorithm.

| **System Organ Class  (SOC)** | **Preferred Terms (PTs)** | **Reporting  Cases** | **ROR (ROR_025_-ROR_975_)** | **PRR (χ^2^)** | **IC (IC**025**-IC**975**)** | **EBGM (EBGM**05**-EBGM**95**)** |
| --- | --- | --- | --- | --- | --- | --- |
| Gastrointestinal disorders | Diarrhoea | 125 | 2.08(1.74-2.48)* | 2.06(68.46)* | 1.04(0.77-1.29)* | 2.05(1.72-2.45) |
|  | Colitis | 79 | 16.09(12.81-20.21)* | 15.93(1047.06)* | 3.92(3.35-4.02)* | 15.13(12.05-19)* |
|  | Vomiting | 49 | 1.07(0.81-1.42) | 1.07(0.25) | 0.1(-0.31-0.51) |  |
|  | Nausea | 42 | 0.70(0.51-0.94) | 0.7(5.56) | -0.52(-0.95--0.07) | 0.7(0.52-0.95) |
|  | Abdominal pain | 33 | 0.92(0.65-1.29) | 0.92(0.25) | -0.12(-0.62-0.38) | 0.92(0.65-1.29) |
|  | Immune-mediated enterocolitis | 26 | 612.5(308.94-1214.34)* | 610.38(4995.24)* | 7.6(3.85-5.29)* | 193.44(97.57-383.5)* |
|  | Pancreatitis | 23 | 3.02(2.00-4.56)* | 3.02(30.68)* | 1.58(0.87-2.06)* | 2.99(1.98-4.52) |
|  | Ascites | 19 | 4.71(2.99-7.41)* | 4.7(54.45)* | 2.21(1.32-2.62)* | 4.64(2.95-7.30)* |
|  | Constipation | 17 | 0.88(0.55-1.41) | 0.88(0.29) | -0.19(-0.86-0.5) | 0.88(0.55-1.41) |
|  | Intestinal obstruction | 17 | 2.10(1.3-3.39)* | 2.1(9.72)* | 1.06(0.29-1.66)* | 2.09(1.3-3.37) |
|  | Enterocolitis | 15 | 32.32(18.93-55.17)* | 32.26(407.68)* | 4.86(2.64-4.16)* | 29.05(17.02-49.58)* |
|  | Enteritis | 15 | 19.6(11.61-33.1)* | 19.56(247.09)* | 4.2(2.39-3.88)* | 18.36(10.87-31)* |
|  | Gastritis | 15 | 2.83(1.70-4.71)* | 2.83(17.54)* | 1.49(0.61-2.06)* | 2.81(1.69-4.67) |
| General disorders and administration site conditions | Death | 290 | 1.71(1.52-1.92)* | 1.68(81.48)* | 0.75(0.57-0.92)* | 1.68(1.49-1.89) |
|  | Pyrexia | 58 | 1.19(0.92-1.54) | 1.18(1.68) | 0.24(-0.14-0.62) | 1.18(0.91-1.53) |
|  | Fatigue | 57 | 0.67(0.52-0.88) | 0.68(8.86) | -0.56(-0.93--0.17) | 0.68(0.52-0.88) |
|  | Asthenia | 45 | 0.91(0.68-1.22) | 0.91(0.43) | -0.14(-0.56-0.29) | 0.91(0.68-1.22) |
|  | General physical health deterioration | 41 | 2.22(1.63-3.02)* | 2.21(27.02)* | 1.14(0.65-1.55)* | 2.2(1.62-2.99) |
|  | Pain | 30 | 0.33(0.23-0.48) | 0.34(39.72)* | -1.57(-2.06--1.02) | 0.34(0.24-0.48) |
|  | Disease progression | 24 | 2.62(1.75-3.92)* | 2.62(23.76)* | 1.38(0.71-1.87)* | 2.6(1.74-3.89) |
|  | Malaise | 24 | 0.45(0.3-0.67) | 0.45(16.12) | -1.15(-1.69--0.54) | 0.45(0.3-0.67) |
|  | Oedema peripheral | 17 | 0.89(0.55-1.43) | 0.89(0.25) | -0.17(-0.85-0.52) | 0.89(0.55-1.43) |
|  | Chest pain | 15 | 0.54(0.33-0.90) | 0.54(5.77) | -0.88(-1.56--0.11) | 0.54(0.33-0.9) |
|  | Drug ineffective | 15 | 0.27(0.16-0.45) | 0.28(28.84) | -1.86(-2.51--1.07) | 0.28(0.17-0.46) |
| Infections and infestations | Pneumonia | 116 | 1.55(1.29-1.86)* | 1.54(22.16)* | 0.62(0.35-0.88)* | 1.54(1.28-1.85) |
|  | Sepsis | 46 | 2.42(1.81-3.24) | 2.41(37.84)* | 1.26(0.8-1.64)* | 2.4(1.79-3.21) |
|  | COVID-19 | 39 | 0.92(0.67-1.26) | 0.92(0.26) | -0.12(-0.57-0.34) | 0.92(0.67-1.26) |
|  | Urinary tract infection | 27 | 0.68(0.46-0.99) | 0.68(4.12) | -0.56(-1.09-0.01) | 0.68(0.47-0.99) |
|  | Encephalitis | 21 | 33.9(21.54-53.33)* | 33.81(596.97)* | 4.92(3.05-4.35)* | 30.29(19.25-47.66)* |
|  | Septic shock | 19 | 3.51(2.23-5.52)* | 3.51(33.63)* | 1.8(0.98-2.28)* | 3.47(2.21-5.47)* |
|  | Cellulitis | 18 | 1.38(0.87-2.2) | 1.38(1.88) | 0.46(-0.23-1.1) | 1.38(0.87-2.19) |
|  | Herpes zoster | 11 | 0.92(0.51-1.66) | 0.92(0.08) | -0.12(-0.95-0.72) | 0.92(0.51-1.66) |
|  | Pneumonia aspiration | 11 | 2.45(1.35-4.43)* | 2.44(9.31)* | 1.28(0.28-1.96)* | 2.43(1.34-4.4) |
|  | Infection | 11 | 0.55(0.30-0.99) | 0.55(4.04) | -0.86(-1.64-0.03) | 0.55(0.31-1) |
|  | Pneumocystis jirovecii pneumonia | 10 | 10.52(5.60-19.79)* | 10.51(82.98)* | 3.35(1.58-3.36)* | 10.17(5.41-19.13)* |
| Respiratory, thoracic and mediastinal disorders | Pneumonitis | 78 | 33.48(26.45-42.38)* | 33.14(2176.32)* | 4.9(4.1-4.79)* | 29.76(23.51-37.67)* |
|  | Dyspnoea | 62 | 0.85(0.66-1.09) | 0.85(1.61) | -0.23(-0.59-0.14) | 0.85(0.66-1.09) |
|  | Interstitial lung disease | 49 | 9.70(7.29-12.90)* | 9.64(367.13)* | 3.23(2.59-3.42)* | 9.35(7.03-12.45)* |
|  | Respiratory failure | 29 | 2.46(1.71-3.55)* | 2.45(24.83)* | 1.29(0.69-1.75)* | 2.44(1.69-3.52) |
|  | Pulmonary embolism | 28 | 1.38(0.95-2.00) | 1.37(2.85) | 0.46(-0.1-0.98) | 1.37(0.95-1.99) |
|  | Pleural effusion | 28 | 2.78(1.92-4.04)* | 2.78(31.56)* | 1.46(0.84-1.92)* | 2.76(1.9-4.01) |
|  | Cough | 28 | 0.75(0.52-1.08) | 0.75(2.38) | -0.42(-0.94-0.13) | 0.75(0.52-1.09) |
|  | Lung disorder | 20 | 2.61(1.68-4.06)* | 2.61(19.63)* | 1.37(0.63-1.9)* | 2.59(1.67-4.03) |
|  | Acute respiratory failure | 16 | 4(2.44-6.55)* | 3.99(35.35)* | 1.98(1.04-2.45)* | 3.95(2.41-6.47)* |
|  | Chronic obstructive pulmonary disease | 16 | 1.62(0.99-2.65) | 1.62(3.78) | 0.69(-0.06-1.34) | 1.62(0.99-2.64) |
|  | Hypoxia | 15 | 3.40(2.04-5.66)* | 3.4(25.08)* | 1.75(0.83-2.28)* | 3.37(2.02-5.61)* |
| Neoplasms benign, malignant and unspecified | Malignant neoplasm progression | 214 | 31.73(27.5-36.61)* | 30.85(5577.04)* | 4.8(4.42-4.84)* | 27.91(24.19-32.2)* |
|  | Metastases to central nervous system | 14 | 9.85(5.78-16.79)* | 9.84(107.39)* | 3.25(1.84-3.36)* | 9.54(5.59-16.26)* |
|  | Acute myeloid leukaemia | 13 | 3.47(2.00-5.99)* | 3.46(22.49)* | 1.78(0.77-2.32)* | 3.43(1.99-5.93) |
|  | Myelodysplastic syndrome | 10.00 | 2.43(1.30-4.53)* | 2.43(8.32)* | 1.27(0.22-1.97)* | 2.41(1.3-4.5) |
|  | Neoplasm malignant | 6 | 0.83(0.37-1.86) | 0.83(0.2) | -0.26(-1.32-0.87) | 0.83(0.37-1.86) |
|  | Basal cell carcinoma | 6 | 0.82(0.37-1.83) | 0.82(0.23) | -0.28(-1.34-0.85) | 0.82(0.37-1.83) |
| Nervous system disorders | Headache | 25 | 0.41(0.28-0.61) | 0.41(21.25) | -1.28(-1.81--0.68) | 0.41(0.28-0.61) |
|  | Dizziness | 24 | 0.49(0.33-0.74) | 0.49(12.51) | -1.02(-1.56--0.41) | 0.49(0.33-0.74) |
|  | Seizure | 22 | 1.05(0.69-1.6) | 1.05(0.06) | 0.08(-0.53-0.68) | 1.05(0.69-1.6) |
|  | Cerebral infarction | 16 | 3.39(2.07-5.55)* | 3.38(26.57)* | 1.75(0.85-2.26)* | 3.36(2.05-5.5)* |
|  | Syncope | 15 | 0.80(0.48-1.33) | 0.8(0.76) | -0.32(-1.03-0.42) | 0.8(0.48-1.33) |
|  | Cerebrovascular accident | 13.00 | 0.44(0.25-0.75) | 0.44(9.4) | -1.19(-1.9--0.36) | 0.44(0.25-0.76) |
|  | Encephalopathy | 12 | 7.47(4.21-13.26)* | 7.46(65.44) | 2.87(1.48-3.11)* | 7.3(4.11-12.95)* |
|  | Neuropathy peripheral | 11 | 1.21(0.67-2.19) | 1.21(0.4) | 0.27(-0.59-1.08) | 1.21(0.67-2.19) |
|  | Hypoaesthesia | 10 | 0.46(0.25-0.85) | 0.46(6.43) | -1.12(-1.92--0.18) | 0.46(0.25-0.85) |
| Injury, poisoning and procedural complications | Off label use | 101 | 2.19(1.80-2.67) | 2.17(63.86)* | 1.11(0.81-1.39)* | 2.16(1.78-2.64) |
|  | Intentional product use issue | 52.00 | 10.58(8.01-13.96) | 10.51(431.61)* | 3.35(2.71-3.52)* | 10.17(7.7-13.42)* |
|  | Fall | 28 | 0.34(0.24-0.5) | 0.34(35.27) | -1.53(-2.04--0.96) | 0.35(0.24-0.5) |
|  | Prescribed underdose | 27 | 9.12(6.21-13.38) | 9.09(188.34)* | 3.14(2.23-3.34)* | 8.83(6.02-12.97)* |
|  | Inappropriate schedule of product administration | 18 | 0.86(0.54-1.36) | 0.86(0.42) | -0.22(-0.87-0.46) | 0.86(0.54-1.37) |
|  | Prescribed overdose | 17 | 16.16(9.91-26.36)* | 16.13(228.16)* | 3.94(2.39-3.79) | 15.31(9.38-24.97)* |
|  | Product use issue | 11 | 1.54(0.85-2.79) | 1.54(2.07) | 0.62(-0.28-1.39) | 1.54(0.85-2.78) |
| Metabolism and nutrition disorders | Decreased appetite | 41 | 2.01(1.47-2.73)* | 2(20.42)* | 1(0.51-1.41)* | 1.99(1.46-2.71) |
|  | Hyponatraemia | 39 | 5.53(4.03-7.6)* | 5.51(141.24)* | 2.44(1.82-2.75)* | 5.42(3.95-7.45)* |
|  | Diabetic ketoacidosis | 37 | 13.72(9.86-19.1)* | 13.66(414.25)* | 3.71(2.83-3.79)* | 13.08(9.39-18.2)* |
|  | Hyperglycaemia | 32 | 7.79(5.48-11.08)* | 7.76(183.64)* | 2.92(2.15-3.17)* | 7.58(5.33-10.78)* |
|  | Dehydration | 32 | 1.75(1.23-2.47)* | 1.74(10.08)* | 0.8(0.26-1.27)* | 1.74(1.23-2.46) |
|  | Diabetes mellitus | 30 | 2.86(1.99-4.09)* | 2.85(35.68)* | 1.5(0.9-1.94)* | 2.83(1.97-4.06) |
|  | Type 1 diabetes mellitus | 26.00 | 27.12(18.12-40.58)* | 27.03(594.72)* | 4.63(3.14-4.3)* | 24.75(16.54-37.03)* |
|  | Fulminant type 1 diabetes mellitus | 22 | 887.99(379.22-2079.34)* | 885.39(4691.17)* | 7.74(3.58-5.18)* | 214.47(91.59-502.22)* |
|  | Hypokalaemia | 19 | 3.24(2.06-5.1)* | 3.23(29.01)* | 1.68(0.88-2.18)* | 3.21(2.04-5.05)* |
|  | Type 2 diabetes mellitus | 15 | 1.68(1.01-2.8)* | 1.68(4.13)* | 0.75(-0.04-1.41) | 1.68(1.01-2.79) |
| Investigations | Weight decreased | 47 | 1.38(1.03-1.84)* | 1.38(4.81)* | 0.46(0.03-0.86)* | 1.37(1.03-1.83) |
|  | Blood creatinine increased | 19 | 2.4(1.53-3.78)* | 2.4(15.39)* | 1.26(0.51-1.81)* | 2.39(1.52-3.75) |
|  | Aspartate aminotransferase increased | 18 | 3.78(2.37-6.02)* | 3.77(36.22)* | 1.9(1.04-2.37)* | 3.74(2.35-5.95)* |
|  | Platelet count decreased | 18 | 1.4(0.88-2.22) | 1.4(2.02) | 0.48(-0.21-1.11) | 1.39(0.88-2.22) |
|  | Alanine aminotransferase increased | 17 | 2.92(1.81-4.72)* | 2.92(21.24)* | 1.54(0.71-2.07)* | 2.9(1.8-4.68) |
|  | Blood bilirubin increased | 12 | 4.87(2.75-8.62)* | 4.86(36.22)* | 2.26(1.08-2.7)* | 4.8(2.71-8.49)* |
|  | Lipase increased | 11 | 12.77(6.98-23.38)* | 12.75(113.99)* | 3.61(1.8-3.51)* | 12.24(6.69-22.41)* |
|  | Gamma-glutamyltransferase increased | 10 | 3.87(2.07-7.22)* | 3.86(20.95)* | 1.94(0.73-2.48)* | 3.83(2.05-7.14)* |
|  | Transaminases increased | 10.00 | 5.52(2.95-10.32)* | 5.51(36.24)* | 2.44(1.07-2.83)* | 5.43(2.9-10.15)* |
|  | Weight increased | 10 | 0.45(0.24-0.84) | 0.45(6.66) | -1.14(-1.94--0.2) | 0.45(0.24-0.84) |
| Skin and subcutaneous tissue disorders | Pemphigoid | 68 | 99.13(75.15-130.76)* | 98.24(4853.32)* | 6.19(4.77-5.55)* | 73.1(55.41-96.42)* |
|  | Rash | 50 | 1.75(1.32-2.31)* | 1.74(15.81)* | 0.8(0.37-1.18)* | 1.74(1.32-2.3) |
|  | Pruritus | 37 | 1.42(1.02-1.96)* | 1.41(4.47)* | 0.5(0.01-0.95)* | 1.41(1.02-1.95) |
|  | Vitiligo | 11 | 57.47(30.04-109.95)* | 57.39(506.32)* | 5.58(2.38-4.19)* | 47.84(25.01-91.53)* |
|  | Erythema | 9 | 0.62(0.32-1.19) | 0.62(2.14) | -0.7(-1.55-0.27) | 0.62(0.32-1.19) |
|  | Skin disorder | 8 | 2.45(1.22-4.91)* | 2.45(6.79)* | 1.28(0.1-2.04)* | 2.43(1.21-4.88) |
|  | Dermatitis psoriasiform | 7 | 36.55(16.63-80.35)* | 36.52(214.08)* | 5.02(1.63-3.8)* | 32.44(14.76-71.32)* |
|  | Stevens-Johnson syndrome | 7.00 | 9.58(4.51-20.36)* | 9.57(51.98)* | 3.22(1.15-3.23)* | 9.29(4.37-19.74)* |
| Musculoskeletal and connective tissue disorders | Arthralgia | 45 | 0.84(0.63-1.13) | 0.84(1.38) | -0.25(-0.67-0.18) | 0.84(0.63-1.13) |
|  | Myalgia | 19 | 1.19(0.76-1.87) | 1.19(0.56) | 0.25(-0.41-0.88) | 1.19(0.76-1.86) |
|  | Back pain | 19 | 0.58(0.37-0.91) | 0.58(5.74) | -0.78(-1.4--0.1) | 0.58(0.37-0.91) |
|  | Muscular weakness | 17 | 0.97(0.60-1.57) | 0.97(0.01) | -0.04(-0.72-0.65) | 0.97(0.6-1.57) |
|  | Arthritis | 15 | 1.24(0.75-2.06) | 1.24(0.7) | 0.31(-0.44-1.01) | 1.24(0.75-2.06) |
|  | Rheumatoid arthritis | 14 | 0.75(0.45-1.27) | 0.75(1.13) | -0.41(-1.13-0.36) | 0.75(0.45-1.28) |
|  | Eosinophilic fasciitis | 12 | 1128.66(318.43-4000.48)* | 1126.86(2699.68)* | 7.82(2.54-4.7)* | 226.17(63.81-801.65)* |
|  | Polyarthritis | 12 | 21.29(11.84-38.31)* | 21.26(215.46) | 4.31(2.18-3.85)* | 19.84(11.03-35.69)* |
|  | Pain in extremity | 11 | 0.23(0.13-0.42) | 0.24(27.37) | -2.08(-2.82--1.15) | 0.24(0.13-0.43) |
| Hepatobiliary disorders | Autoimmune hepatitis | 20 | 27.69(17.48-43.86)* | 27.62(467.34)* | 4.66(2.89-4.21)* | 25.24(15.94-39.98)* |
|  | Drug-induced liver injury | 19 | 14.95(9.42-23.72)* | 14.91(234.21)* | 3.83(2.43-3.76)* | 14.21(8.95-22.55)* |
|  | Hepatitis | 16 | 8.39(5.1-13.81)* | 8.38(100.98)* | 3.03(1.81-3.23)* | 8.17(4.96-13.43)* |
|  | Hepatic function abnormal | 13.00 | 5.76(3.32-9.98)* | 5.75(50)* | 2.5(1.3-2.86)* | 5.65(3.26-9.8)* |
|  | Cholecystitis | 13 | 3(1.73-5.18)* | 2.99(17.07)* | 1.57(0.6-2.16)* | 2.97(1.72-5.13) |
|  | Cholangitis | 13 | 14.16(8.11-24.73)* | 14.14(151.17)* | 3.76(2.04-3.62)* | 13.51(7.74-23.6)* |
|  | Immune-mediated hepatitis | 12.00 | 564.33(211.74-1504.03)* | 563.43(2245.75)* | 7.56(2.57-4.65)* | 188.48(70.72-502.32)* |
|  | Immune-mediated hepatic disorder | 12 | 1128.66(318.43-4000.48)* | 1126.86(2699.68)* | 7.82(2.54-4.7)* | 226.17(63.81-801.65)* |
|  | Jaundice | 12 | 4.03(2.28-7.13)* | 4.02(26.9)* | 1.99(0.89-2.5)* | 3.98(2.25-7.04)* |
|  | Hepatic failure | 11 | 4.26(2.35-7.72)* | 4.25(26.95)* | 2.07(0.89-2.57)* | 4.2(2.32-7.63)* |
|  | Cholangitis sclerosing | 10 | 32.05(16.66-61.68)* | 32.01(269.8)* | 4.85(2.11-3.94)* | 28.85(14.99-55.51)* |
| Cardiac disorders | Cardiac failure | 30 | 2.08(1.45-2.98)* | 2.08(16.66)* | 1.05(0.48-1.52)* | 2.07(1.44-2.97) |
|  | Atrial fibrillation | 21 | 1.32(0.86-2.02) | 1.32(1.59) | 0.39(-0.24-0.99) | 1.31(0.86-2.02) |
|  | Myocardial infarction | 19 | 0.53(0.34-0.84) | 0.53(7.77) | -0.9(-1.52--0.22) | 0.53(0.34-0.84) |
|  | Pericardial effusion | 18 | 4.92(3.09-7.85)* | 4.91(55.17)* | 2.28(1.34-2.68)* | 4.85(3.04-7.73)* |
|  | Myocarditis | 14 | 17.48(10.18-30.01)* | 17.45(204.48)* | 4.04(2.25-3.79)* | 16.49(9.61-28.31)* |
|  | Cardiac arrest | 11 | 0.89(0.49-1.6) | 0.89(0.16) | -0.17(-0.99-0.68) | 0.89(0.49-1.61) |
| Renal and urinary disorders | Acute kidney injury | 71 | 2.72(2.15-3.44)* | 2.71(75.85)* | 1.43(1.05-1.74)* | 2.69(2.13-3.4)* |
|  | Renal failure | 31 | 1.46(1.02-2.08)* | 1.46(4.42)* | 0.54(0.01-1.03)* | 1.45(1.02-2.07) |
|  | Tubulointerstitial nephritis | 20 | 13.35(8.52-20.92)* | 13.32(217.66)* | 3.67(2.38-3.68)* | 12.76(8.15-20)* |
|  | Proteinuria | 13 | 6.4(3.69-11.1)* | 6.39(57.83)* | 2.65(1.41-2.97)* | 6.27(3.62-10.87)* |
|  | Nephritis | 12 | 30.78(16.96-55.88)* | 30.73(311.25)* | 4.8(2.34-4.02)* | 27.81(15.32-50.48)* |
|  | Renal impairment | 12 | 1.35(0.77-2.39) | 1.35(1.11) | 0.44(-0.41-1.2) | 1.35(0.77-2.39) |
| Blood and lymphatic system disorders | Anaemia | 53 | 1.6(1.22-2.1) | 1.6(11.76)* | 0.67(0.26-1.05)* | 1.59(1.21-2.09) |
|  | Thrombocytopenia | 44 | 4.17(3.09-5.62)* | 4.15(103.76)* | 2.04(1.51-2.37)* | 4.1(3.04-5.53)* |
|  | Febrile neutropenia | 17 | 6.62(4.09-10.71)* | 6.61(79.05)* | 2.7(1.62-3)* | 6.48(4-10.48)* |
|  | Leukopenia | 13 | 2.61(1.51-4.51)* | 2.61(12.76)* | 1.37(0.44-1.99)* | 2.59(1.5-4.48) |
|  | Lymphadenopathy | 12 | 2.22(1.26-3.92)* | 2.22(7.97)* | 1.14(0.21-1.82)* | 2.21(1.25-3.9) |
|  | Neutropenia | 11 | 1.12(0.62-2.02) | 1.12(0.13) | 0.16(-0.69-0.98) | 1.12(0.62-2.02) |
|  | Immune thrombocytopenia | 10 | 9.5(5.06-17.84)* | 9.49(73.45)* | 3.2(1.51-3.28)* | 9.21(4.9-17.3)* |
| Endocrine disorders | Adrenal insufficiency | 55 | 67.56(50.31-90.74)* | 67.07(2891.88)* | 5.76(4.37-5.22)* | 54.37(40.48-73.02) |
|  | Hypothyroidism | 42 | 10.43(7.66-14.21)* | 10.38(343.51)* | 3.33(2.6-3.5)* | 10.05(7.38-13.68)* |
|  | Hypophysitis | 23 | 464.24(238.8-902.52)* | 462.82(4010.45)* | 7.46(3.65-5.16)* | 175.74(90.4-341.66)* |
|  | Adrenocorticotropic hormone deficiency | 14 | 0(0-0) | 0(3944.04) | 8.14(2.78-4.89)* | 282.71(0-0) |
|  | Hyperthyroidism | 12 | 7.05(3.98-12.51)* | 7.04(60.71)* | 2.79(1.43-3.06)* | 6.9(3.89-12.23)* |
|  | Inappropriate antidiuretic hormone secretion | 10 | 9.83(5.23-18.47)* | 9.82(76.52)* | 3.25(1.53-3.31)* | 9.52(5.07-17.89)* |
| Vascular disorders | Hypotension | 39 | 1.92(1.4-2.63)* | 1.92(17.01)* | 0.93(0.44-1.36)* | 1.91(1.39-2.62) |
|  | Hypertension | 19 | 0.74(0.47-1.16) | 0.74(1.72) | -0.43(-1.06-0.23) | 0.74(0.47-1.16) |
|  | Vasculitis | 8 | 7.28(3.61-14.69)* | 7.27(42.18)* | 2.83(1.1-3.06)* | 7.11(3.52-14.35)* |
|  | Embolism | 7 | 8.36(3.94-17.74)* | 8.36(44.03)* | 3.03(1.06-3.14)* | 8.14(3.84-17.28)* |
|  | Deep vein thrombosis | 6 | 0.39(0.18-0.88) | 0.39(5.61) | -1.34(-2.3--0.12) | 0.39(0.18-0.88) |
|  | Haemorrhage | 6 | 0.44(0.2-0.97) | 0.44(4.34) | -1.19(-2.16-0.02) | 0.44(0.2-0.98) |
|  | Thrombosis | 5 | 0.42(0.18-1.02) | 0.42(3.94) | -1.24(-2.27-0.09) | 0.42(0.18-1.02) |
| Psychiatric disorders | Confusional state | 27 | 1.48(1.01-2.16)* | 1.48(4.14)* | 0.56(-0.01-1.08) | 1.47(1.01-2.15) |
|  | Mental status changes | 9 | 2.64(1.37-5.09)* | 2.64(9.05)* | 1.39(0.25-2.09)* | 2.62(1.36-5.05) |
|  | Delirium | 9 | 3.54(1.83-6.83)* | 3.54(16.17)* | 1.81(0.57-2.4)* | 3.5(1.82-6.77) |
|  | Depression | 9 | 0.28(0.14-0.53) | 0.28(17) | -1.85(-2.65--0.83) | 0.28(0.14-0.53) |
|  | Insomnia | 6 | 0.26(0.12-0.57) | 0.26(12.89) | -1.95(-2.89--0.7) | 0.26(0.12-0.57) |
|  | Anxiety | 6 | 0.13(0.06-0.29) | 0.13(34.41) | -2.92(-3.82--1.63) | 0.13(0.06-0.3) |
| Eye disorders | Cataract | 7 | 0.72(0.34-1.5) | 0.72(0.79) | -0.48(-1.45-0.59) | 0.72(0.34-1.51) |
|  | Uveitis | 7 | 3.49(1.66-7.36)* | 3.49(12.29)* | 1.79(0.37-2.43)* | 3.46(1.64-7.29) |
|  | Dry eye | 6 | 1.96(0.88-4.37) | 1.96(2.8) | 0.96(-0.32-1.88) | 1.95(0.87-4.36) |
|  | Visual acuity reduced | 5 | 1.12(0.46-2.69) | 1.12(0.06) | 0.16(-1.05-1.31) | 1.12(0.46-2.69) |
|  | Vision blurred | 5 | 0.42(0.17-1.00) | 0.42(4.06) | -1.26(-2.29-0.07) | 0.42(0.17-1.01) |

**Supplementary Table 4 Signal strength of delayed irAEs at Preferred Terms (PT).** ROR, Reporting odds ratio; CI, Confidence interval; ROR_025_, The lower limit of 95% CI of the ROR; ROR_975_, The upper limit of 95% CI of the ROR; PRR, Proportional reporting ratio; χ, Chi-squared; IC, Information component; IC_025_, The lower limit of 95% CI of the IC; IC_975_, The upper limit of 95% CI of the IC; EBGM, Empirical Bayesian geometric mean; EBGM_05_, The lower limit of 95% CI of EBGM; EBGM_95_, The upper limit of 95% CI of EBGM. *Indicates statistically significant signals in algorithm.

| SOC | Reporting cases in females | Reporting cases in males | ROR | ROR**_025_** | ROR**_975_** | χ**^2^** | P |
| --- | --- | --- | --- | --- | --- | --- | --- |
| Gastrointestinal disorders | 299 | 536 | 1.05 | 0.9 | 1.22 | 0.37 | 0.54 |
| General disorders and administration site conditions | 274 | 535 | 0.95 | 0.82 | 1.11 | 0.4 | 0.53 |
| Infections and infestations | 218 | 429 | 0.94 | 0.8 | 1.12 | 0.43 | 0.51 |
| Respiratory, thoracic and mediastinal disorders | 171 | 399 | 0.79 | 0.65 | 0.95* | 6.43 | 0.01* |
| Nervous system disorders | 165 | 286 | 1.08 | 0.89 | 1.32 | 0.63 | 0.43 |
| Neoplasms benign, malignant and unspecified | 158 | 291 | 1.02 | 0.83 | 1.24 | 0.02 | 0.88 |
| Injury, poisoning and procedural complications | 147 | 262 | 1.05 | 0.85 | 1.29 | 0.22 | 0.64 |
| Metabolism and nutrition disorders | 144 | 258 | 1.05 | 0.85 | 1.29 | 0.17 | 0.68 |
| Investigations | 121 | 243 | 0.93 | 0.74 | 1.16 | 0.44 | 0.51 |
| Skin and subcutaneous tissue disorders | 120 | 233 | 0.96 | 0.77 | 1.2 | 0.12 | 0.73 |
| Blood and lymphatic system disorders | 110 | 132 | 1.58 | 1.22* | 2.05 | 12.36 | 0.00* |
| Musculoskeletal and connective tissue disorders | 99 | 199 | 0.93 | 0.72 | 1.19 | 0.37 | 0.54 |
| Cardiac disorders | 99 | 159 | 1.17 | 0.91 | 1.51 | 1.45 | 0.23 |
| Hepatobiliary disorders | 93 | 175 | 0.99 | 0.77 | 1.28 | 0.00 | 0.96 |
| Renal and urinary disorders | 77 | 180 | 0.79 | 0.6 | 1.04 | 2.82 | 0.09 |
| Endocrine disorders | 75 | 142 | 0.99 | 0.74 | 1.31 | 0.01 | 0.92 |
| Vascular disorders | 54 | 112 | 0.9 | 0.65 | 1.25 | 0.41 | 0.52 |
| Psychiatric disorders | 40 | 55 | 1.36 | 0.91 | 2.06 | 2.22 | 0.14 |
| Eye disorders | 29 | 64 | 0.84 | 0.54 | 1.31 | 0.56 | 0.45 |
| Immune system disorders | 21 | 18 | 2.19 | 1.16* | 4.12 | 6.22 | 0.01* |
| Pregnancy, puerperium and perinatal conditions | 19 | 7 | 5.1 | 2.14 | 12.15 | 16.78 | 0.00 |
| Surgical and medical procedures | 14 | 28 | 0.93 | 0.49 | 1.78 | 0.04 | 0.83 |
| Ear and labyrinth disorders | 6 | 19 | 0.59 | 0.23 | 1.48 | 1.3 | 0.25 |
| Reproductive system and breast disorders | 4 | 11 | 0.68 | 0.22 | 2.13 | 0.44 | 0.5 |
| Congenital, familial and genetic disorders | 2 | 0 | - | - | - | - | 0.12 |
| Social circumstances | 2 | 3 | 1.25 | 0.21 | 7.46 | - | 1 |
| Product issues | 0 | 9 | - | - | - | 3.41 | 0.06 |

**Supplementary Table 5** Sex differences of delayed irAEs at SOC level. SOC, Organ System Class; ROR, Reporting odds ratio; CI, Confidence interval; ROR_025_, The lower limit of 95% CI of the ROR; ROR_975_, The upper limit of 95% CI of the ROR; χ, Chi-squared; IC, Information component; *Indicates statistically significant signals in algorithm.
